# Stromal cell‐derived factor‐1 downregulation contributes to neuroprotection mediated by CXC chemokine receptor 4 interactions after intracerebral hemorrhage in rats

**DOI:** 10.1111/cns.14400

**Published:** 2023-08-24

**Authors:** Yu Wu, Zhuwei Zhang, Xiaoou Sun, Jing Wang, Haitao Shen, Xue Sun, Zhong Wang

**Affiliations:** ^1^ Department of Neurosurgery & Brain and Nerve Research Laboratory The First Affiliated Hospital of Soochow University Su Zhou China; ^2^ Department of Neurosurgery Linyi People's Hospital Linyi China; ^3^ Department of Emergency Medicine The First Affiliated Hospital of Soochow University Su Zhou China

**Keywords:** AMD3100, CXC chemokine receptor 4, ICH, neuroprotection, stromal cell‐derived factor‐1

## Abstract

**Aim:**

Stromal cell‐derived factor‐1 (SDF‐1) and CXC chemokine receptor 4 (CXCR4) have a substantial role in neuronal formation, differentiation, remodeling, and maturation and participate in multiple physiological and pathological events. In this study, we investigated the role of SDF‐1/CXCR4 in neural functional injury and neuroprotection after intracerebral hemorrhage (ICH).

**Methods:**

Western blot, immunofluorescence and immunoprecipitation were used to detect SDF‐1/CXCR4 expression and combination respectively after ICH. TUNEL staining, Lactate dehydrogenase assay, Reactive oxygen species assay, and Enzyme‐linked immunosorbent assay to study neuronal damage; Brain water content to assay brain edema, Neurological scores to assess short‐term neurological deficits. Pharmacological inhibition and genetic intervention of SDF‐1/CXCR4 signaling were also used in this study.

**Results:**

ICH induced upregulation of SDF‐1/CXCR4 and increased their complex formation, whereas AMD3100 significantly reduced it. The levels of TNF‐α and IL‐1β were significantly reduced after AMD3100 treatment. Additionally, AMD3100 treatment can alleviate neurobehavioral dysfunction of ICH rats. Conversely, simultaneous SDF‐1/CXCR4 overexpression induced the opposite effect. Moreover, immunoprecipitation confirmed that SDF‐1/CXCR4 combined to initiate neurodamage effects.

**Conclusion:**

This study indicated that inhibition of SDF‐1/CXCR4 complex formation can rescue the inflammatory response and alleviate neurobehavioral dysfunction after ICH. SDF‐1/CXCR4 may have applications as a therapeutic target after ICH.

## Graphical abstract

SDF‐1/CXCR4 in brain tissue of rats increase significantly and induce brain damage after ICH. The effects of SDF‐1 may be associated with the inhibition of the inflammatory response induced by combination with CXCR4, and SDF‐1/CXCR4 may be a potential target for treatment of ICH.

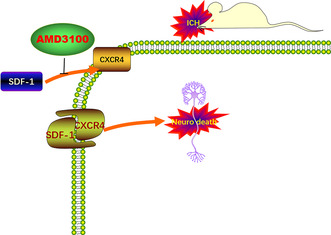

## 1 INTRODUCTION

Intracerebral hemorrhage (ICH) is a major cause of mortality and disability among comparatively healthy individuals worldwide, and it can result in a serious decline in the quality of life for patients who survive.^1^ Currently, an effective treatment modality for ICH is not available, with some patients resorting to hematoma evacuation, which is not satisfactory. 75% patients survive with comorbidities, including varying degrees of motor, sensory, language and other higher nerve function defects. Although significant progress has been made in studying mechanisms, clinical effective treatment of brain injury after ICH, whether it can significantly improve the prognosis of ICH is still not available. The pathophysiological mechanisms caused by ICH are remarkably complicated and involve a string of events such as neuroinflammation; oxidative stress; cell apoptosis, which results in increased blood–brain barrier (BBB) permeability; cerebral edema; and neurological impairment, all of which largely determine the prognosis and outcome of ICH patients, Therefore, extensive research efforts on the pathogenesis and protective effects of ICH are urgently needed.^2^, ^3^, ^4^

Stromal cell‐derived factor‐1 (SDF‐1) and its specific receptor, chemokine receptor 4 (CXCR4), are widely expressed in the developing and mature brain.^5^, ^6^, ^7^, ^8^ They are important regulators of cell migration and mainly involved in cell proliferation, differentiation, and repair. They also participate in several pathological processes such as inflammation, immune activation, tissue dysplasia, tumor development, and stem cell development.^9^, ^10^, ^11^, ^12^, ^13^, ^14^ The SDF‐1 and CXCR4 complex has a particularly important role in neurogenesis, as it mediates proliferation of neurogenitors, mobilizes neural hematopoietic stem cells; and is involved in angiogenesis, brain development, neurodegeneration, and neurogenesis. SDF‐1/CXCR4 is involved in some nervous system diseases, including acute cerebral ischemia, the vasculogenesis and remodeling of cerebral arteriovenous malformation, and neurodegenerative diseases such as Parkinson's disease.^15^, ^16^, ^17^, ^18^, ^19^ In neurodegeneration that occurs after stroke, astrocytes in the vicinity of the damaged area of the brain are activated and secreted chemokine SDF‐1, which acts on chemokine expressed by endogenous neural progenitor cells and stimulates their targeted migration to the damaged area. The SDF‐1/CXCR4 is also involved in several inflammatory processes and diseases including infections, autoimmune disorders, pulmonary fibrosis. SDF‐1 is believed to regulate neuronal excitability and synaptic transmission. SDF‐1 not only enhances GABA and glutamate activities of 5‐hydroxytryptamine neurons, but also enhances the proliferation and migration of neuronal stem cells, and the damage of these mechanisms has been proved to be associated with neurodegenerative diseases.

After ICH, there is a significant increase in neurogenesis in the subventricular and subgranular regions of the brain.^20^, ^21^ The damaged tissue release SDF‐1 and matrix metalloproteinases (MMPs) to repair this damage and promote the regeneration of neurons.^22^, ^23^, ^24^, ^25^ On the other hand, neuroinflammation after ICH contributed significantly to brain injury, and SDF‐1/CXCR4 is involved in inflammatory processes. Therefore, the exact role of SDF‐1/CXCR4 on ICH is still uncertain and needs further study. In this study, we hypothesized that neuronal injury after ICH maybe caused by pathological activation of SDF‐1/CXCR4 and that selective inhibition of SDF‐1/CXCR4 may delay the progression of neuronal injury after ICH. We aimed to investigate SDF‐1/CXCR4 expression and activity in a rat model of ICH and to further examine the effects and potential mechanism of SDF‐1/CXCR4 in brain injury and neuroprotection after ICH.

## 2 MATERIALS AND METHODS

### 2.1 Animals

The animal experimental agreements were approved by the Experimental Animals Committee of the First Affiliated Hospital of Soochow University and complied with the ARRIVE (Animal Research: Reporting of In Vivo Experiments) guidelines. We strived to minimize the number of rats used and their pain. Adult male Sprague–Dawley rats weighing 280‐300 g were purchased from the Animal Center of the Chinese Academy of Sciences. All rats were housed in a quiet environment maintained at 18–22°C and humidity with 12 h light/dark cycles. Animals had free access to food and water.

### 2.2 Experimental design

In our study, all rats were randomly assigned (using a random‐number table) before the experiments. The researchers of the experiments were blind to the random assignments. All the data were labeled with random numbers according to their randomly assigned numbers. Statistics were performed unblinded in groups.

This study consists of three experimental parts. In the first part, we detected the expression of SDF‐1 and CXCR4 among the Normal group, Sham group and the ICH rats at different time points (six rats in each group, these rats were randomly assigned, Figure S1B). The eight time points examined were 1 h, 3 h, 6 h, 12 h, 24 h, 48 h, 72 h, and 168 h after the operation. These time courses were used to determine the proper timepoint in the experiments described below. Expression of SDF‐1 and CXCR4 was detected by western blot and immunofluorescence. At the appropriate time point, the rats were anesthetizated by gaseous anesthesia and the brain tissue were stored in liquid nitrogen, and the right hemisphere basal ganglia tissue, which included the hematoma and surrounding areas, were separated, and collected for experiments (Figure S1A).

In the second part, we used 72 rats randomly divided into six groups with an average of 12 rats per group (Figure S1C). The six groups were the Sham group, ICH group, ICH + negative‐control siRNA (ICH + Si‐control) group, ICH + SDF‐1 siRNA (ICH + Si‐SDF‐1) group, ICH + Vehicle group, and ICH + AMD3100 group. For 6 rats in each group, the neurological scoring was carried out at 24 h after surgery, then these 6 rats were anesthetizated as described above, their brain tissue were isolated for edema measurements. For the other 6 rats per group, the right hemisphere basal ganglia tissue were collected as described above. These collected tissue were then used to determine oxidative stress levels, the level of neuronal apoptosis, and fluorescence detection. Sample sources of inflammatory factors are serum and cerebrospinal fluid (CSF), which should be specified. CSF was also used to test for Lactate dehydrogenase (LDH), and brain tissue were also used for Reactive oxygen species (ROS), western blot, immunoprecipitation and immunofluorescence.

In the third part, we tested the effect of upregulation of SDF‐1 or CXCR4 on ICH (Figure S1D). We used 144 rats, which were randomly divided into eight groups with an average of 18 rats per group. The eight groups were the Sham group, the ICH group, the ICH + Vehicle group, the ICH + recombinant SDF‐1 protein (r‐SDF‐1) group, the ICH + AMD3100 + r‐SDF‐1 group, the ICH + Vector group, the ICH + Adenovirus‐CXCR4 (Ad‐CXCR4) group, and the ICH + Ad‐CXCR4+ Si‐SDF‐1 group respectively. The details of samples collection and experiments design were same as described above in the second experiment. For additional 6 rats, from 21 to 26 days after ICH modeling, the Morris Water Maze (MWM) place navigation test and spatial probe test were performed to assess the spatial‐dependent cognition deficits of the rats in each group.

### 2.3 ICH modeling

The ICH model protocol was carried out as described below in our previous study.^26^ For details, please see Supplementary Materials.

### 2.4 Behavioral testing

Behavioral testing was performed at 24 h after ICH as described in Experimental design section. Briefly, a double‐blind investigator completed the neurological defect and revised the Garcia score report.^26^ In general, this analysis covers seven parameters: spontaneous movement activity, body proprioception, vibration touch, symmetry of limb movement, lateral rotation, forelimb out‐stretching, and climbing ability respectively. Scores for each subtest ranged from 0 to 3, with a combined maximum score of 21. This test was performed by two technicians who were blinded to the experimental groupings.

### 2.5 Brain water content

Brain edema is a phenomenon of concern in the injured hemisphere after ICH. We measured the brain water content by using the dry/wet method, as described previously.^26^ For details, please see Supplementary Materials.

### 2.6 BBB Injury

BBB permeability was assessed based on albumin extravasation.^26^ The change of albumin level in brain tissue can be used as an indicator to judge the degree of BBB injury, western blot was used to detect the level of albumin in the brain tissue of rats in each group.

### 2.7 Enzyme‐linked immunosorbent assay (ELISA)

CSF and blood samples were collected from individual rats, the blood was centrifuged and the supernatant was taken, then the CSF and serum were centrifuged at 2–6°C for about 20 min (3000 rpm). Levels of TNF‐α and IL‐1β in these samples were examined by an ELISA assay with a specific kit (Cloud Clone, China). This experiment was conducted in according to the manufacturer's instructions. Values are expressed as picograms per milliliter of protein (pg/mg protein).^26^

### 2.8 Brain tissue lysates and immunoprecipitation

For lysate preparation, all steps were carried out on ice. Harvested brain tissue (surrounding the hematoma in the ipsilateral hemisphere) was lysed in 5 vol lysis buffer with protease inhibitors. The mixture was incubated on ice for 30 min, followed by centrifugation in a 4°C centrifuge for 15 min at 20,000 × g. The supernatant was then collected for further analysis.

For immunoprecipitation, a volume of lysate corresponding to 1 mg protein was then incubated overnight at 4°C with 20 μL primary beads covalently bound to a primary antibody. The mixture was then washed five times with lysis buffer and 60 μL elution buffer (0.2 M glycine, pH 2.8) for 5 min. The eluates were then rapidly neutralized with 6 μL of 1 M Tris–HCl (pH 7.4) and used for Western blot.^10^

### 2.9 Western blot analysis

The Western blot analysis protocol was carried out as described below in a previous study.^27^ For details, please see Supplementary Materials.

### 2.10 Immunofluorescence staining

The Immunofluorescence staining protocol was carried out as described below in a previous study.^24^ For details, please see Supplementary Materials.

### 2.11 TUNEL staining

The TUNEL staining protocol was carried out as described below in our previous study.^26^ For details, please see Supplementary Materials.

### 2.12 LDH activity assay

LDH activity in CSF supernatants was quantified using a specific LDH assay according to the manufacturer's instructions (Nanjing Jiancheng Bioengineering Institute). For details, please see Supplementary Materials.

### 2.13 ROS activity assay

ROS activity in brain tissue supernatants was quantified using a specific ROS Detection assay kit according to the manufacturer's instructions (Red Fluorescence, abcam). For details, please see Supplementary Materials.

### 2.14 Drug administration

#### 2.14.1 AMD3100 Treatment

To disrupt the binding of SDF‐1 and CXCR4, we injected 1 mg/kg AMD3100 (1 μL PBS solution containing 0.3 mg AMD3100 for each rat, Sigma Aldrich) into the lateral ventricle of rats immediately after 0.5 h of ICH modeling and simultaneously injected 1 μL solvent into its control group in the same way.^26^ The coordinates of intraventricular injection were 1.0 mm in the lateral direction relative to the bregma, 0.5 mm in the posterior direction, and 2.5 mm in depth.

#### 2.14.2 Construction of siRNAs

To knock down SDF‐1 expression, the specific siRNA for SDF‐1 were provided by GenScript. siRNA construction is done by specialized companies (GenScript). Transfection of siRNA in the rat brain was performed using Entranster‐in vivo RNA transfection reagent (Engreen) according to the manufacturer's instructions. According to the manufacturer's instructions, 5 μL si‐SDF‐1 was dissolved in 10 μL in vivo siRNA transfection reagent. The solution was mixed for 15 min at room temperature. Finally, 15 μL Entranster‐in vivo‐mixture was injected into the intracerebroventricularly 48 h before ICH.

#### 2.14.3 Stereotaxic adenovirus injection

CXCR4 was overexpressed via Ad‐CXCR4 (provided by GenScript and stored at −80°C) transfection. 1 μL Ad‐CXCR4 according to manufacturer's instructions (1:1:E+13 V.g/ml) was stereotactically injected into lateral ventricles for each rats. The coordinates from Bregma are 1.0 mm behind, side 1.8 mm, depth 2.4 mm. Leave the needle for 5 min before injecting Ad‐CXCR4 and slowly pull it out. We established ICH models 3 weeks after Ad‐CXCR4 injection.

#### 2.14.4 Administration of r‐SDF‐1

We carried out administration of the r‐SDF‐1 in rat brains. 20 μL/kg r‐SDF‐1 solution was injected intraventricularly 48 h before the ICH procedure.

### 2.15 Morris water maze test

The Rats were tested in a circular tank with a diameter of 180 cm and a depth of 50 cm, with a platform placed 2 cm below the surface of the water. The learning and memory impairments were evaluated after ICH at 21 to 26 days. The incubation period, swimming path, swimming distance to the hidden platform and swimming speed are all automatically recorded by the camera. The number of times the rat swam within the quadrant of the removed platform was automatically recorded. This test was performed by two technicians who were blinded to the experimental groupings.

### 2.16 Statistical analysis

SPSS 19.0 statistics software (SPSS Inc.) were used for data analysis. All data were presented as the mean ± SEM. The normal distribution of data sets was analyzed by the Kolmogorov–Smirnov test. Data groups (two groups) with normal distributions were compared using the two‐tailed unpaired Student's *t*‐test, and the Mann Whitney *U*‐test was used for nonparametric data. Data obtained using the Morris Water Maze test were analyzed using two‐way Repeated ANOVA. Differences were considered statistically significant at a value of *p* < 0.05. A detailed description of the statistical analysis was presented in Table S1.

## 3 RESULTS

### 3.1 Time course of SDF‐1 and CXCR4 expression after ICH

**FIGURE 1:**
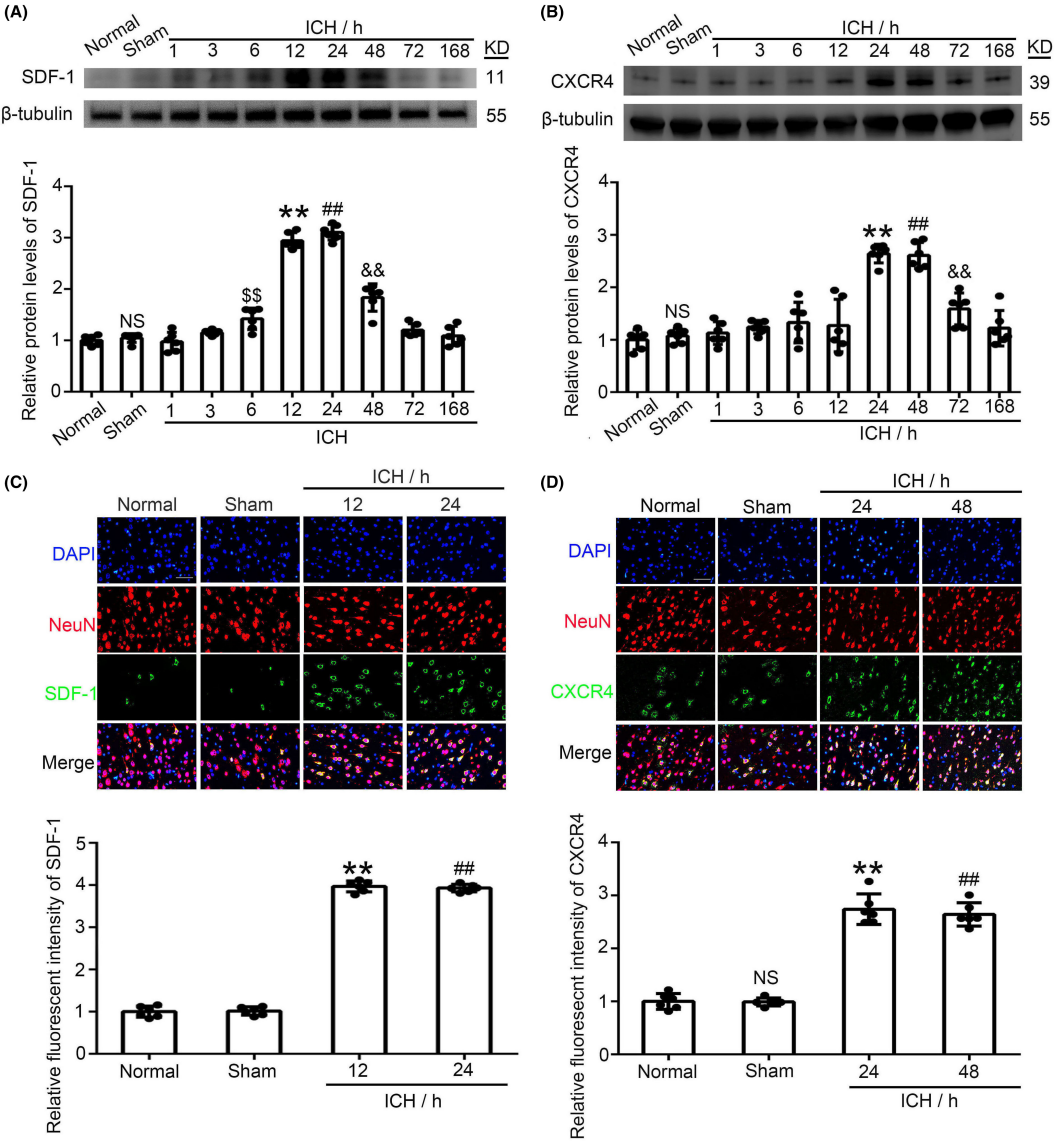
Expression of SDF‐1 (A) and CXCR4 (B) at different times was measured by western blot after ICH. Immunofluorescence staining was used to evaluate changes in SDF‐1 (C) and CXCR4 (D) in neurons after ICH. Green was the SDF‐1/CXCR4 and red was NeuN, and Blue was DAPI. It was found that both can co‐locate of the neuron. Tissue sections were taken from the basal ganglia region of the right hemisphere (site of the ICH and its surrounding tissue). Data are shown as the mean ± SEM; $$*p* < 0.01, **p < 0.01, ##*p* < 0.01, &&*p* < 0.01 vs. Sham. Scale bar = 50 μm.

The brain region of interest is surrounding the hematoma in the ipsilateral hemisphere, which did not include the area the needle inserted. We analyzed the expression of SDF‐1 and CXCR4 after ICH to determine the appropriate timepoint for subsequent experiments. SDF‐1 expression increased at ~12 h in the ICH rats as compared with Sham rats. The peak value was reached at 12 h and 24 h after ICH, and a downward trend was noted after 48 h. After 168 h after ICH, the level had basically returned to Sham group (Figure 1A,C). The results of immunofluorescence showed that SDF‐1 and CXCR4 were both expressed on the cell and could co‐locate with neuronal marker NeuN (Figure 1E–H). To further confirm the cellular localization of CXCR4, the immunofluorescence analysis with CXCR4 and GFAP/Iba1 (a marker of astrocytes and microlia, respectively) was also performed in this study. Undoubltly, the levels of CXCR4 were increased in brain tissue after ICH. However, we found that CXCR4‐positive cells were not mainly co‐localization with GFAP/Iba1‐positive cells (Figure S2A,B).

### 3.2 Effect of Si‐SDF‐1 and AMD3100 intervention after ICH

**FIGURE 2:**
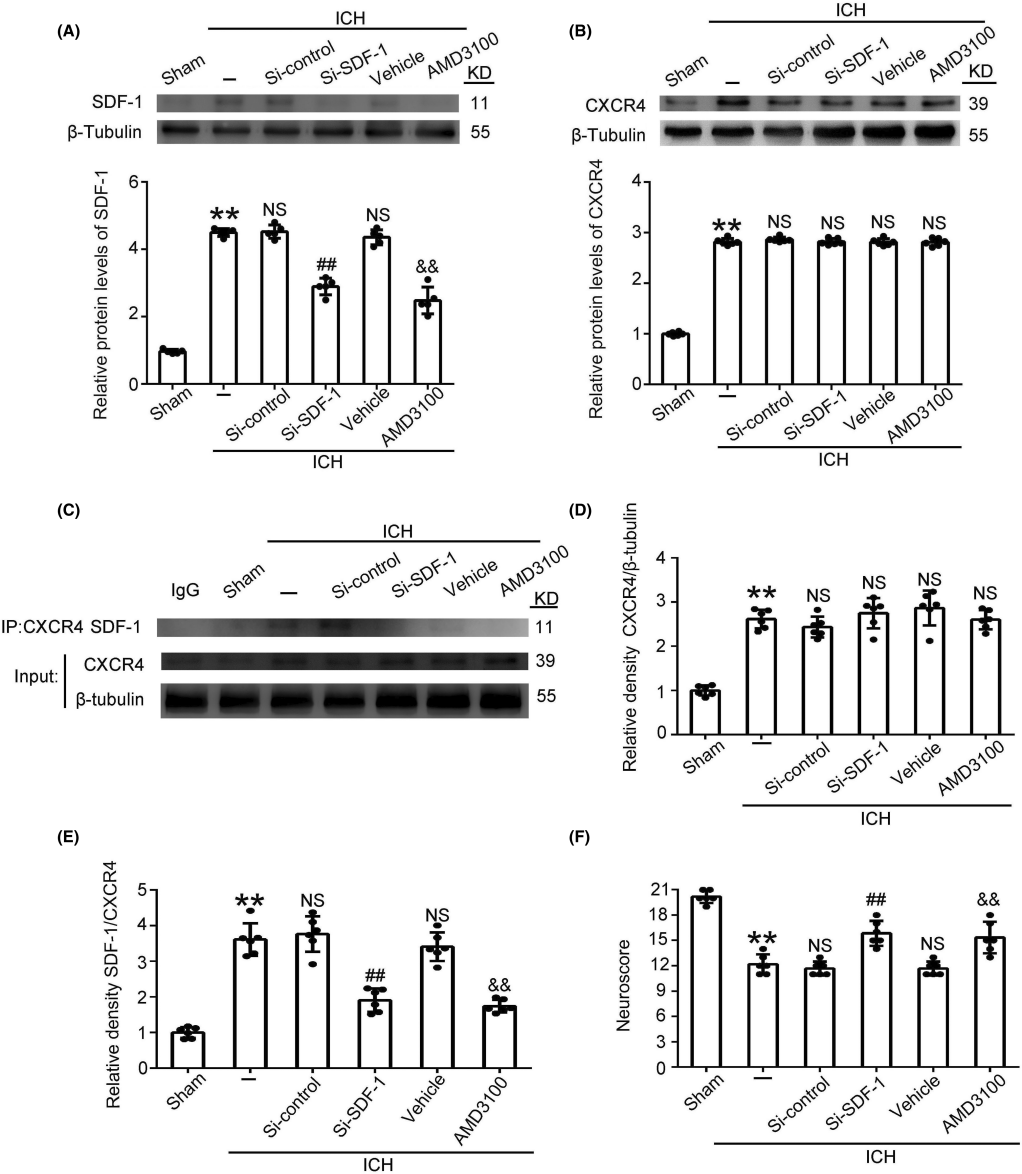
Analysis of changes in SDF‐1 (A) and CXCR4 (B) expression under different treatments. In rats from the ICH group, level of SDF‐1 was decreased by siRNA (Si‐SDF‐1) intervention. Brain tissue lysates after ICH were subjected to immunoprecipitation (IP) with anti‐CXCR4 antibody, analyzed the level of SDF‐1 by western blot (C). The interaction between SDF‐1/CXCR4 was blocked in the presence of AMD3100. Relative density CXCR4/β‐tubulin and SDF‐1/CXCR4 were analyzed by statistical graphs (D, E). The modified Garcia test was representative of neurobehavioral scores in the experimental groups (F). Data are shown as the mean ± SEM; **p < 0.01 vs. Sham; ##*p* < 0.01 vs. ICH + Si‐control; &&*p* < 0.01 vs. ICH + Vehicle.

To examine the role of SDF‐1 and to characterize the involvement of the SDF‐1 and CXCR4 in ICH, we used different approaches to disrupt expression of SDF‐1 (siRNA) and SDF‐1 and CXCR4 binding (AMD3100). We found that when SDF‐1 expression was knockdown by siRNA, the expression of the CXCR4 did not decrease relative to levels in its control group. Meanwhile, the AMD3100 does reduce the amount of SDF‐1, but not CXCR4 (Figure 2A,B). We found similar results by immunofluorescence staining and treatment with AMD3100 did not affect the expression of CXCR4. (Figure S3).

We further analyzed interactions between SDF‐1 and CXCR4 after ICH by immunoprecipitation. When SDF‐1 was knockdown by siRNA, its level interacted with CXCR4 was significantly decreased compared with that in its control group (Figure 2C–E). When treatment with AMD3100, the level of SDF‐1 interacted with CXCR4 was also significantly decreased than that in its control group. These results indicated that SDF‐1 and CXCR4 interacted during ICH, while both Si‐SDF‐1 and AMD3100 treatment inhibited the binding of SDF‐1 and CXCR4. To detect the neurological functions of SDF‐1 and CXCR4 in ICH, the neuroscore test was performed. After ICH, the neuroscore of rats were significantly reduced compared with those in Sham group; but inhibition of the SDF‐1 and CXCR4 interaction by AMD3100, similarly to reduction of SDF‐1 levels by siRNA, substantially reversed this decline in neuronal function. (Figure 2F).

### 3.3 Effect of Si‐SDF‐1 and AMD3100 treatment on brain injury after ICH

In both serum and CSF, there were similar changes in levels of TNF‐α and IL‐1β. As compared with Sham group, TNF‐α and IL‐1β levels in the ICH group were significantly higher, whereas reduced levels of SDF‐1 or treatment with AMD3100 was associated with a significant reduction in the levels of TNF‐α and IL‐1β (Figure S4A–D). Meanwhile, we determined the level of oxidative stress by ROS activity of the injured brain tissue and further collected the CSF to detect LDH activity. As compared with Sham group, the ROS and LDH levels of the ICH group were both higher, whereas reduction of SDF‐1 expression by siRNA significantly reduced the ROS and LDH levels after ICH. Similar results were noted in AMD3100 treatment group (Figure S4E,F).

**FIGURE 3:**
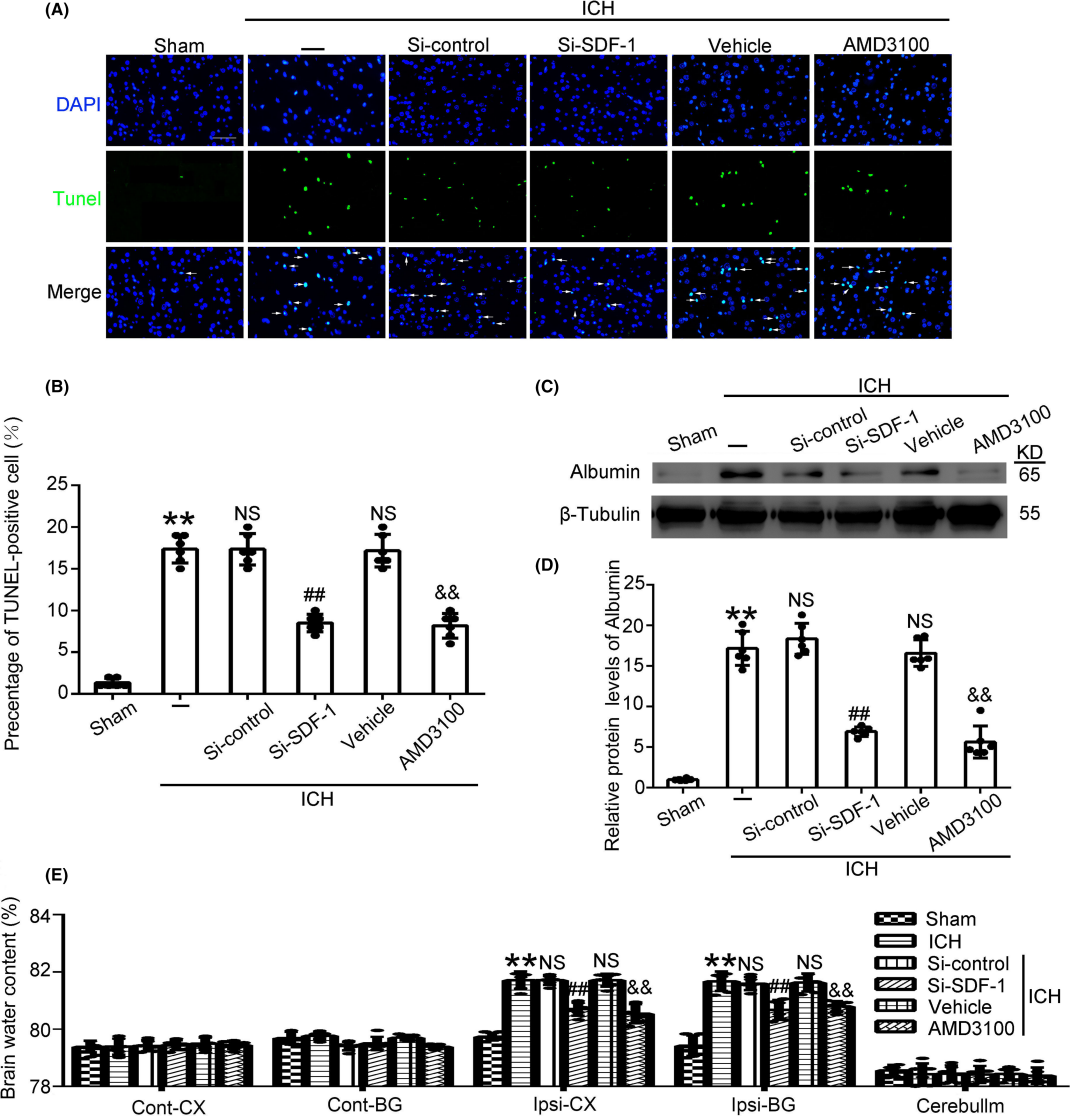
TUNEL assay for cell apoptosis in the basal ganglia region of ICH rats brain after different treatments (A, B). Green fluorescence indicated TUNEL staining and blue fluorescence indicated nuclei. Scale bar = 50 μm. The protein levels of Albumin in brain tissue of rats in various treatments groups were examined (C, D) and the neuroprotective effect of SDF‐1 inhibition on brain water content (E). CX, cerebral cortex; BG, basal ganglia (site of the ICH); cont, contralateral (uninjured) hemisphere; ipsi, ipsilateral (injured) hemisphere. Data are shown as the mean ± SEM, **p < 0.01 vs. Sham; ##*p* < 0.01 vs. ICH + Si‐control; &&*p* < 0.01 vs. ICH + Vehicle.

To examine the role of SDF‐1 in apoptosis, we performed TUNEL staining at 24 h after ICH. Cell apoptosis was obviously increased in the ICH group as compared with Sham group. The number of TUNEL‐positive cells was reduced after SDF‐1 siRNA treatment, which is consistent with the effect of the AMD3100 (Figure 3A,B). In addition, the integrity of the BBB was determined by the level of albumin leakage into the cortical brain tissue that surrounded the site of injury. In comparison with Sham group, we found that the amount of albumin present increased substantially after ICH, which was reversed in SDF‐1 knockdown group and AMD3100 treatment group (Figure 3C,D).

Additionally, there was a significant increase in brain water content in the lpsi‐CX and Ipsi‐BG regions in ICH rats as compared with Sham rats. When SDF‐1 was knockdown or AMD3100 treatment, brain water content in the ipsilateral basal ganglia and cortical regions was alleviated to some extent (Figure 3E).

### 3.4 Simultaneous upregulation of SDF‐1/CXCR4 on brain injury after ICH

**FIGURE 4:**
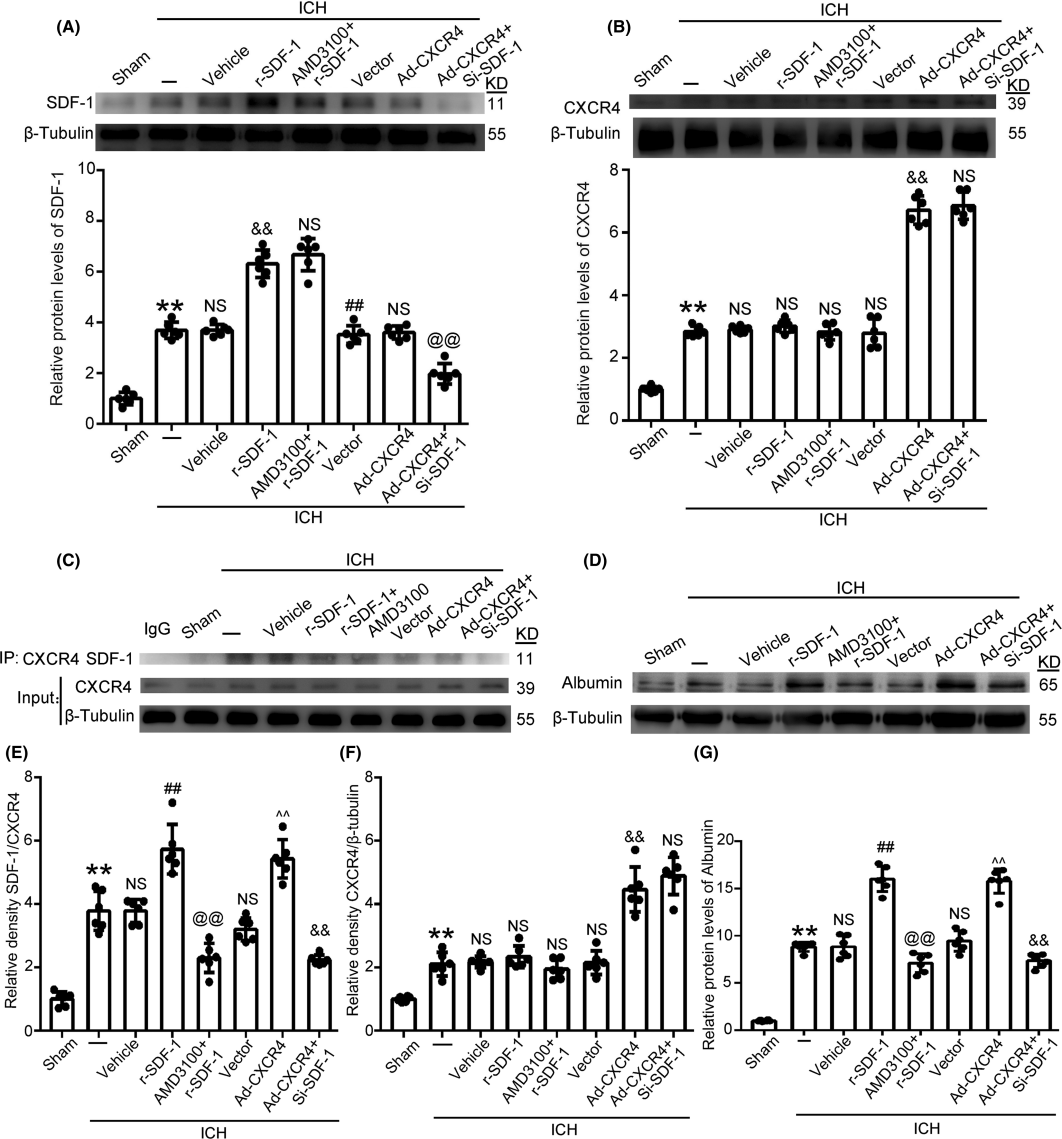
Analysis of SDF‐1 and CXCR4 expression levels under different treatments after ICH (A‐B). The levels of SDF‐1 and CXCR4 were increased by r‐SDF‐1 administration. Brain tissue lysates were subjected to immunoprecipitation (IP) with anti‐CXCR4 antibody, analyzed the levels of SDF‐1 by western blot (C). Relative density of SDF‐1/CXCR4 and CXCR4/β‐tubulin were analyzed (E, F). The protein levels of Albumin in brain tissue of rats in various treatments groups were examined (D, G). Data are shown as the mean ± SEM; **p < 0.01 vs. Sham; ##*p* < 0.01 vs. ICH + Vehicle; @@*p* < 0.01 vs. ICH + r‐SDF‐1; ^^*p* < 0.01 vs. ICH + Vector; &&*p* < 0.01 vs. ICH + Ad‐CXCR4.

Furthermore, we administered the exogenous recombinant SDF‐1 and the levels of CXCR4 were overexpressed via transfected adenovirus vector (Ad‐CXCR4). The recombinant protein increased the level of SDF‐1, we confirmed an expected increase in expression of SDF‐1 after ICH relative to its control group. Increased SDF‐1 had no effect on the expression of CXCR4 after ICH. Meanwhile, although the expression of CXCR4 was successfully increased by overexpression, the level of SDF‐1 has not similar increasing. The results of immunofluorescence also confirmed these. (Figure 4A,B and Figure S5).

Additionally, when administered SDF‐1 was combined with AMD3100 treatment, the results of IP indicated that the levels of SDF‐1 interacted with CXCR4 was decreased due to AMD3100 inhibited the binding of SDF‐1 and CXCR4. When CXCR4 was overexpressed, the levels of SDF‐1 interacted with CXCR4 also increased. Conversely, when both r‐SDF‐1 and AMD3100 administration, or CXCR4 overexpressed and SDF‐1 was knockdown by siRNA, IP showed a decrease in SDF‐1 level interacted with CXCR4 (Figure 4C,E,F). Next, when r‐SDF‐1 administered, the level of albumin was increased, whereas when SDF‐1 was increased with the presence of AMD3100, increased albumin level was alleviated to some extent. Albumin level was also increased when CXCR4 overexpressed alone. When both CXCR4 overexpressed and SDF‐1 knockdown, the increased level of albumin was alleviated to some extent (Figure 4D,G). When SDF‐1 or CXCR4 upregulated, levels of TNF‐α and IL‐β in both the serum and CSF were significantly increased relative to levels in their control groups. When both r‐SDF‐1 and AMD3100 administration, or CXCR4 overexpressed and SDF‐1 knockdown, the increased levels of TNF‐α and IL‐β were alleviated to some extent (Figure S6).

**FIGURE 5:**
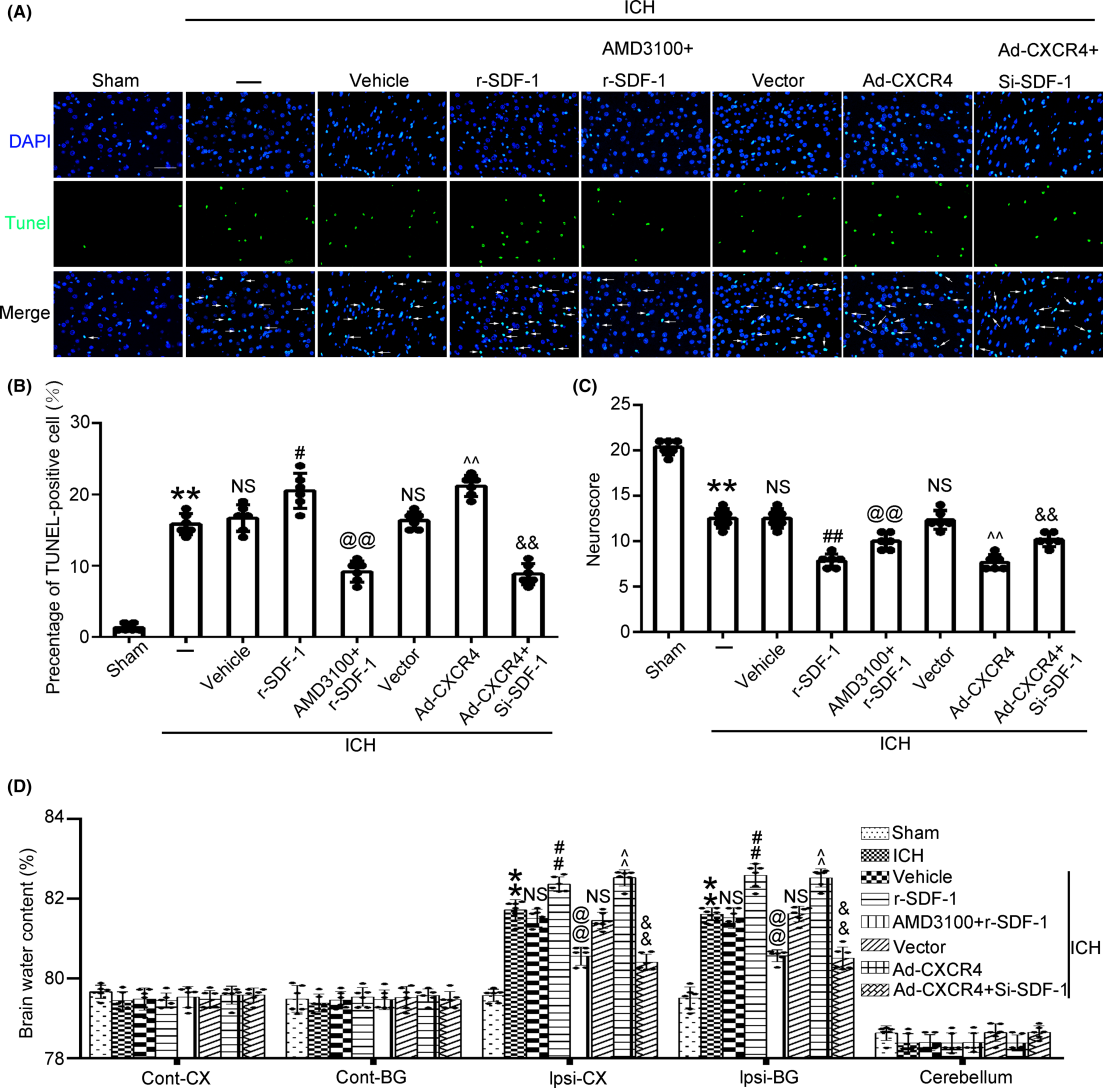
TUNEL assay in the basal ganglia region of ICH rats brain after different treatments (A, B). Green fluorescence indicated TUNEL staining and blue fluorescence indicated nuclei. Scale bar = 50 μm. The effect of SDF‐1/CXCR4 upregulation on neurological function score (C) and brain water content (D) after ICH were examined. CX, cerebral cortex; BG, basal ganglia (site of the ICH); cont, contralateral (uninjured) hemisphere; ipsi, ipsilateral (injured) hemisphere. Data are shown as the mean ± SEM; **p < 0.01 vs. Sham; #*p* < 0.05, ##*p* < 0.01 vs. ICH + Vehicle; @@*p* < 0.01 vs. ICH + r‐SDF‐1; ^^*p* < 0.01 vs. ICH + Vector; &&*p* < 0.01 vs. ICH + Ad‐CXCR4.

Furthermore, the number of TUNEL‐positive cells in brain tissue was increased after r‐SDF‐1 administration, and the same effect was showed in the CXCR4 overexpressed group. When both r‐SDF‐1 and AMD3100 administration, or CXCR4 overexpressed and SDF‐1 knockdown, the increased number of TUNEL‐positive cells was significantly alleviated (Figure 5A,B). Neuroscore of rats were significantly decreased when SDF‐1 or CXCR4 upregulated. However, both r‐SDF‐1 and AMD3100 administration, or CXCR4 overexpressed and SDF‐1 knockdown, the reduced neuroscore were remarkedly alleviated (Figure 5C). Meanwhile, when SDF‐1 or CXCR4 upregulated, brain edema was aggravated; while both r‐SDF‐1 and AMD3100 administration, or CXCR4 overexpressed and SDF‐1 knockdown, the increased brain water content was obviously alleviated (Figure 5D).

**FIGURE 6:**
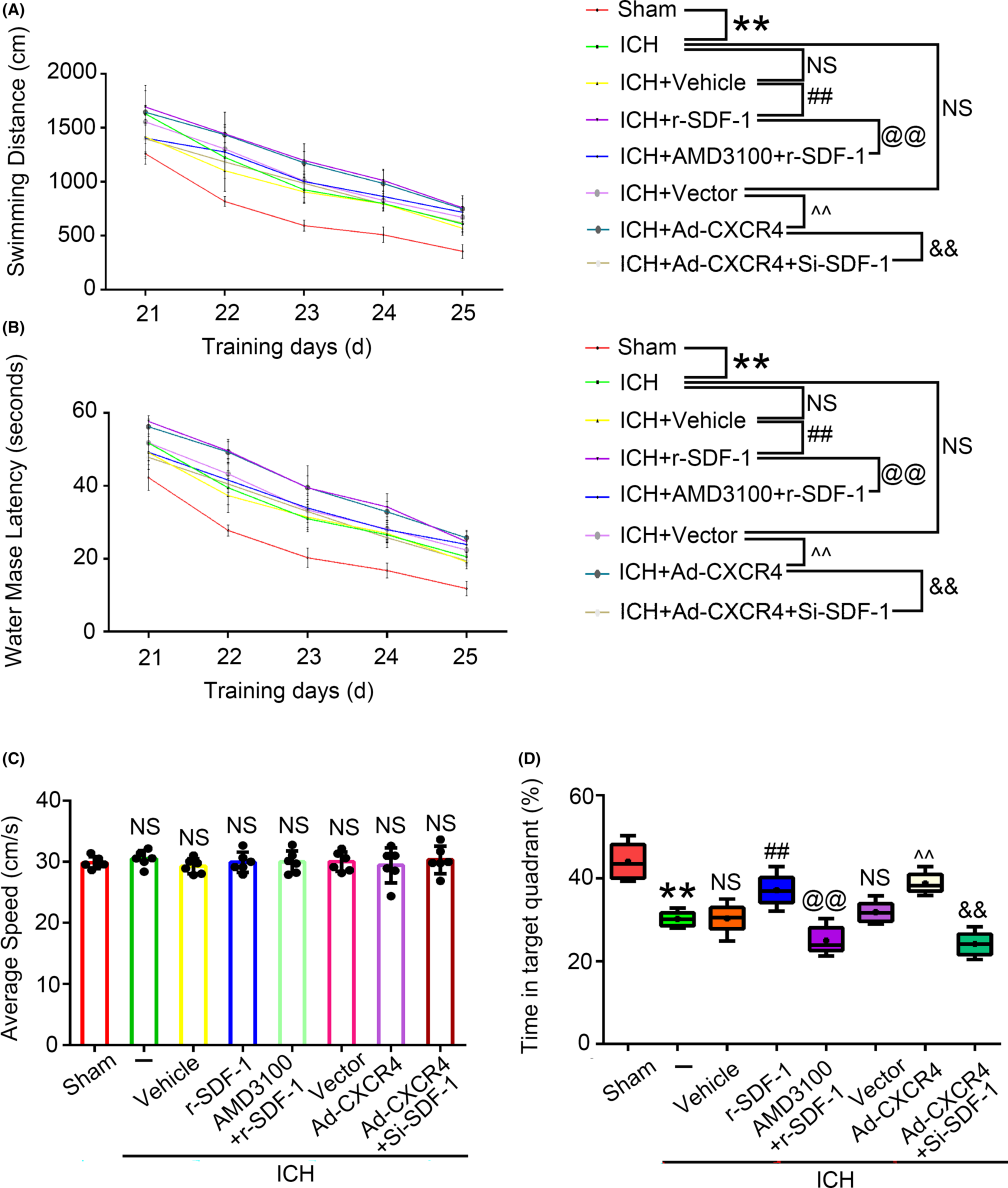
Effects of upregulation of SDF‐1 and CXCR4 treatment on neurological behavioral impairment in ICH. (A) Evaluation of swimming distance in each group. (B) Alterations in water mase latency in each group. (C) Average speed in each group after ICH. (D) Time in target quadrant. All quantitative data are presented as mean ± SEM, **P < 0.01 vs. Sham; ##P < 0.01 vs. ICH + Vehicle; @@*p* < 0.01 vs. ICH + r‐SDF‐1; ^^*p* < 0.01 vs. ICH + Vector; &&*p* < 0.01 vs. ICH + Ad‐CXCR4.

We also evaluated the motor and memory functions of rats after ICH by using Morris water maze test. The SDF‐1 or CXCR4 upregulated rats took longer time and traveled farther distance to complete the task than that in its control group, which suggested that spatial cognition and learning ability were more severely impaired. However, both r‐SDF‐1 and AMD3100 administration, or CXCR4 overexpressed and SDF‐1 knockdown, the longer latency and swimming distance of rats were significantly alleviated compared with their control groups (Figure 6A–C). Also, the proportion of the time spent in the target quadrant was increased, indicating that memory was also improved (Figure 6D).

## 4 DISCUSSION

ICH is an extremely serious stroke event with high disability rate and mortality. It has a poor quality of life and seriously affects the quality of life and long‐term prognosis of patients. ICH brings heavy economic burden to patients and their families. Therefore, it is great importance to explore the neuroprotective effect after ICH. At present, inflammation and cell death are the hot spots in the study of the mechanism of ICH. It is believed that the activation of neuroinflammation after ICH is related to the apoptosis of neurons.^27^, ^28^, ^29^

SDF‐1, a cytokine of mesenchymal origin, and its receptor, CXCR4, belong to the CXC class of chemokines and to the CXCR class G protein‐coupled receptor super‐family, respectively. SDF‐1/CXCR4 has an important role in a variety of biological processes such as immunity, inflammation, embryonic development, and organogenesis, as well as in tumor growth or metastasis, HIV disease, and WHIM (warts, hypogammaglobulinemia, infections, and myelokathexis) syndrome.^3^, ^29^, ^30^, ^31^ CXCR4 is expressed on the surface of embryonic cells, hematopoietic stem cells, and nerve cells. The role of SDF‐1/CXCR4 in promoting repair of damaged tissue has also been recently demonstrated.^32^, ^33^ Previous studies have shown that SDF‐1 may be involved in regulating the ability of repair cells to mobilize, proliferate, and adhere by binding to CXCR4.^8^, ^34^, ^35^ Other studies have shown that SDF‐1 treatment stimulates the ability of proliferative repair cells to adhere.^7^, ^36^ These results indicated that SDF‐1 not only binds to and activates the receptor but also plays a positive role by up‐regulating CXCR4 expression. However, the molecular mechanism by which SDF‐1 stimulates CXCR4 expression requires further investigation.

In this study, we found that SDF‐1 expression increased rapidly when ICH occurs, and the expression reached its peak at 24–48 h, and gradually decreased to normal about a week after ICH. Similarly, the SDF‐1 receptor CXCR4 was activated synchronously, and the time window was the same. When SDF‐1 expression was knockdown, the neurofunction damage can be improved. Meanwhile, by using an inhibitor of SDF‐1/CXCR4 combination, AMD3100, SDF‐1 and CXCR4 interaction was significantly inhibited, although the expression of SDF‐1 and CXCR4 was not significantly affected, which also showed same neuroprotective effect. According to the third part of the experiment, we believed that SDF‐1/CXCR4 was bound to each other to play a neuroprotective role, rather than a single protein. Because when SDF‐1 protein was knocked down, CXCR4 overexpression had no effect after ICH compared to its control group. On the contrary, it was the same when CXCR4 was knocked down and SDF‐1 was overexpressed.

SDF‐1 has been specifically demonstrated in secondary brain injury and neuronal apoptosis after ICH. The detailed mechanism is not very clear, we suggested the following possibilities. (i) SDF‐1 binds to its receptor CXCR4 and activates multiple signaling pathways through G‐protein coupling.^37^ When SDF‐1 binds its receptor CXCR4, it can induce Janus kinases (JAK2 and JAK3) through the coupling effect of its Gαi protein and initiate the STAT signaling pathway, which includes four subtypes (STAT1/2/3/5). This signaling pathway is associated with proliferation, differentiation, migration, survival, and apoptosis.^38^, ^39^, ^40^ (ii) The SDF‐1 binds CXCR4, by binding to the Gαi and Gβγ subunits, activates the phosphatidylinositol‐3‐kinase (PI3K) signaling pathway and its major downstream pathways, such as Akt, mitogen‐activated protein kinase (MAPK), and the nuclear transcription factor NF‐κB pathways.^41^, ^42^, ^43^ (iii) The Gβγ subunit triggers the activation of phosphatidylinositol‐4,5‐bisphosphate (PIP2), diacylglycerol (DAG), and inositol triphosphate (IP3) through phospholipase C (PLC) and the mobilization of calcium ions (Ca^2+^), and increased Ca^2+^ levels activate proline‐rich tyrosine kinase (PYK2), which induces the activation of ERK1/2.^44^, ^45^

Our study did not involve clinical patients, so it is worth continuing to combine with clinical research. Meanwhile, many ICH clinical patients are middle‐aged and elderly; in the current study, the animals used were all young male SD rats, which does not fully simulate the clinical condition. Last but not least, SDF‐1 and CXCR4 were interacted with each other in the cell membrane peaked at 24 h after ICH, the underlying molecular mechanisms need to be further studied.^46^, ^47^, ^48^, ^49^

Taken together, our results demonstrated that SDF‐1/CXCR4 confers important effects in brain injury in a rat model of ICH. The effects of SDF‐1 combined with CXCR4 may be associated with the activation of the inflammatory response after ICH, and SDF‐1/CXCR4 may be a potential target for treatment of ICH.

## AUTHOR CONTRIBUTIONS

All authors had full access to all the data in the study and take responsibility for the integrity of the data and the accuracy of the data analysis. Conceptualization, Z.W. and X.S, methodology, X.S., X.S, software, H.S, formal analysis, J.W, resources, H.S, data curation, Z.Z, writing‐original draft preparation, Y.W, writing—review and editing, Y.W., X.S; visualization, X.S, supervision, Z.W, project administration, Z.W, funding acquisition, Z.W.

## FUNDING INFORMATION

This work was supported by the National Natural Science Foundation of China under Grant (81,873,741, 82,171,309); Doctoral Training Program the Natural Science Foundation of the First Affiliated Hospital of Soochow University (BXQN202142).

## CONFLICT OF INTEREST STATEMENT

The authors declare no conflict of interest.

## INFORMED CONSENT STATEMENT

Informed consent was obtained from all subjects involved in the study.

## Supporting information


Appendix S1.

Table S1.

## Data Availability

The data presented in this study are available on request from the corresponding author.
