# Supplementary material for: Stromal cell‐derived factor‐1 downregulation contributes to neuroprotection mediated by CXC chemokine receptor 4 interactions after intracerebral hemorrhage in rats
Source: CNS Neurosci Ther. 2023 Aug 24;30(2):e14400. doi: 10.1111/cns.14400 (PMC10848108; doi:10.1111/cns.14400)
Supplement: Supplementary file 1 — Appendix S1. [file CNS-30-e14400-s001.zip › cns14400-sup-0001-figures.docx]

**Stromal cell-derived factor-1 downregulation contributes to neuroprotection mediated by CXC chemokine receptor 4 interactions after intracerebral hemorrhage in rats**

Yu Wu^1#^, Zhuwei Zhang^3#^, Xiaoou Sun^1^, Jing Wang^1^, Haitao Shen^1^, Xue Sun^2*^ Zhong Wang^1*^,

1Department of Neurosurgery& Brain and Nerve Research Laboratory, The First Affiliated Hospital of Soochow University, Su Zhou, Jiangsu, PR, China

2Department of Emergency Medicine, The First Affiliated Hospital of Soochow University, Su Zhou, Jiangsu, PR, China

3 Department of Neurosurgery, Linyi People’s Hospital, Linyi, Shandong, PR, China

*Authors to whom correspondence should be addressed: [wangz8761@163.com](mailto:wangzhong8761@163.com) (Zhong Wang), Tel.: +86-13306208761, Department of Neurosurgery& Brain and Nerve Research Laboratory, The First Affiliated Hospital of Soochow University, Su Zhou, Jiangsu, PR, China;

[sunxue1987@suda.edu.cn](mailto:sunxue1987@suda.edu.cn) (Xue Sun), Tel.: +86-18662509688, Department of Emergency Medicine, The First Affiliated Hospital of Soochow University, Su Zhou, Jiangsu, PR, China

# These authors contributed equally to this work.

**Supplemental Materials and Methods**

**2.3 ICH modeling**

Briefly, the rats were firstly anesthetized and attached to a stereotactic device. Next, we placed the rat supine on a device with a heating pad and kept it at about 34-36°C. A microsyringe was inserted through burr holes such that the tip was in the basal ganglia (3.5 mm lateral to the midline, 0.2 mm posterior to the bregma, and 5.5 mm ventral to the cortical surface). After the microsyringe was in place, autologous blood (100 μl total volume) was injected slowly over a 5-min duration, and the needle was left in place for an additional 5min. Bone wax was then used to plug the burr holes and prevent CSF and blood from blood vessels from leaking across the midline. Finally, we sewed up the scalp and put the rat back in its cage, where it had free access to food and water. Sham-operated animals underwent equivalent injections with normal saline without injury. All animals were individually housed after the operation. A general picture of the results of ICH model is shown in Figure 1A.

**2.5 Brain water content**

Briefly, the brain was separated after the isolated after each rat was sacrificed by excessive anesthesia, the brain was divided into ipsilateral and contralateral hemi-spheres, and the wet weight of each hemisphere was immediately determined. Each hemisphere sample was then baked in an oven at 100°C for 72h to determine its dry weight. The percentage of dry brain moisture (%) for each hemisphere was calculated with equation below: [(wet weight-dry weight)/wet weight] × 100%.

**2.9 Western blot analysis**

The supernatant from each lysate tube was individually collected, and the protein concentration of each sample was measured with the standard BCA method (Thermo Fisher, USA). We then used 10% or 15% SDS-polyacrylamide gels for electrophoresis according to the molecular weight of the target protein. Equal amounts of protein (20 μg) were loaded for each sample. The gels were transferred to polyvinylidene difluoride (PVDF) membranes (Millipore, USA). The membrane was next blocked with 5% nonfat milk in TBST for 1h at room temperature. The membranes were incubated with the appropriate primary antibodies diluted in 5% bovine serum albumin (BSA) overnight at 4°C, followed by washing in TBST and then incubation with the appropriate horseradish peroxidase-linked secondary antibodies diluted in 5% BSA for 2h at room temperature. The membranes were washed with TBST and visualized using enhanced chemiluminescence detection (Clinx Science Instruments Co, China). Relative quantities of protein levels were determined using ImageJ software (National Institutes of Health, USA).

**2.10 Immunofluorescence staining**

The isolated brains were frozen as described above and sectioned 15 μm thick. Five isometric profiles (15 for each group) were obtained at the bregma level between -2.12 mm and -4.80 mm, and then the cortex of each brain was immunostained and quantified. The sections were then fixed with 4% paraformaldehyde in paraffin for 24h at 4°C, the paraffin section coronal part (15 μm) of the brain was permeated in 0.1% Triton X-100 for 0.5h and then was blocked in 5% BSA for 1h at room temperature. The sections were incubated overnight at 4°C with the following individual primary antibodies: rabbit monoclonal anti-CXCR4 (1:200; Abcam, USA); rabbit polyclonal anti-SDF-1 (1:200; Abcam, USA), mouse anti-NeuN (1:200; Millipore, USA), mouse anti-GFAP (1:200; Abcam, USA), mouse anti-Iba1 (1:200; Abcam, USA). The sections were then washed with PBST five times for 10min each and then were incubated with appropriate fluorescently labeled secondary antibodies for 2h at room temperature. Negative control staining was performed by omitting the primary antibody (data not shown). Finally, the slides were stained and reacted with 4’,6-diamidino-2-phenylindole dihydrochloride (DAPI) (Southern Biotech, USA) for 10min. The brain sections were observed and analyzed by a fluorescence microscope (Nikon, Japan). The target regions were surrounding the hematoma, which did not include the area the needle inserted. An investigator blind to the design of the study did the immunofluorescence observation. The images were analyzed using ImageJ software (National Institutes of Health, USA).

**2.11 TUNEL staining**

A terminal deoxynucleotidyl transferase (TdT) dUTP Nick-End Labeling (TUNEL) assay was performed to detect apoptotic cells in the region around the hematoma of rats after ICH. The paraffin sections were dehydrated and hydrated, and then 20 µg/ml DNase I-free protein kinase was dripped onto each sample for 15-30min at 35-37°C. The sections were then flushed with PBS five times to remove the protein kinase and were transferred to the TUNEL reaction mixture and incubated for 1h at 37°C. The labeled sections were gently rinsed with PBS five times for 10min each time and were then cover-slipped with an anti-fading mounting medium containing DAPI. Images of the sections were obtained with the confocal fluorescence microscope (DMi8, Leica, Germany). The target regions were surrounding the hematoma, which did not include the area the needle inserted. An investigator blind to the design of the study did the observation. The average number of TUNEL-positive cell per square millimeter, was then counted for each sample.

**2.12 LDH activity assay**

LDH kit refers to lactate dehydrogenase detection kit, which is a 2-[4-Iodophenyl]-3-[4-nitrophenyl]-5-phenyl-tetrazolium chloride (INT) color reaction catalyzed by diaphorase. We set up the experimental wells, the standard wells, the control wells, the sample wells and the blank wells. Then add 50 μl of the substrate mixture to each well, cover the plate with tin foil or an opaque box to block out light, and incubate at room temperature for 30min. Each well was filled with 50 μl of the stop solution, the large bubbles were punctured with a syringe needle, and the absorbance was measured at 490nm within 1h after the stop solution was added.

**2.13 ROS activity assay**

ROS Detection assay kit (Red Fluorescence, abcam, USA) is a sensitive fluorometric one-step assay to detect intracellular ROS, such as superoxide ROS red dye to quantify: the dye is cell-permeable fluorescent signal (Ex/Em=520/605nm). The brain tissue was taken and lysated as described above. The DHE was diluted with PBS at 1:200 (the prepared probe should be placed away from light), and about 3 ml was added to each hole of the 24-well plate (the tissue can be completely infiltrated). Incubate in a 37°C for 20min. Wash with PBS three times to fully remove DCFH-DA that did not enter the tissue. Fluorescence signal was observed at 535nm excitation wavelength and 610nm emission wavelength by fluorescence microplate.

**Supplemental Figures**


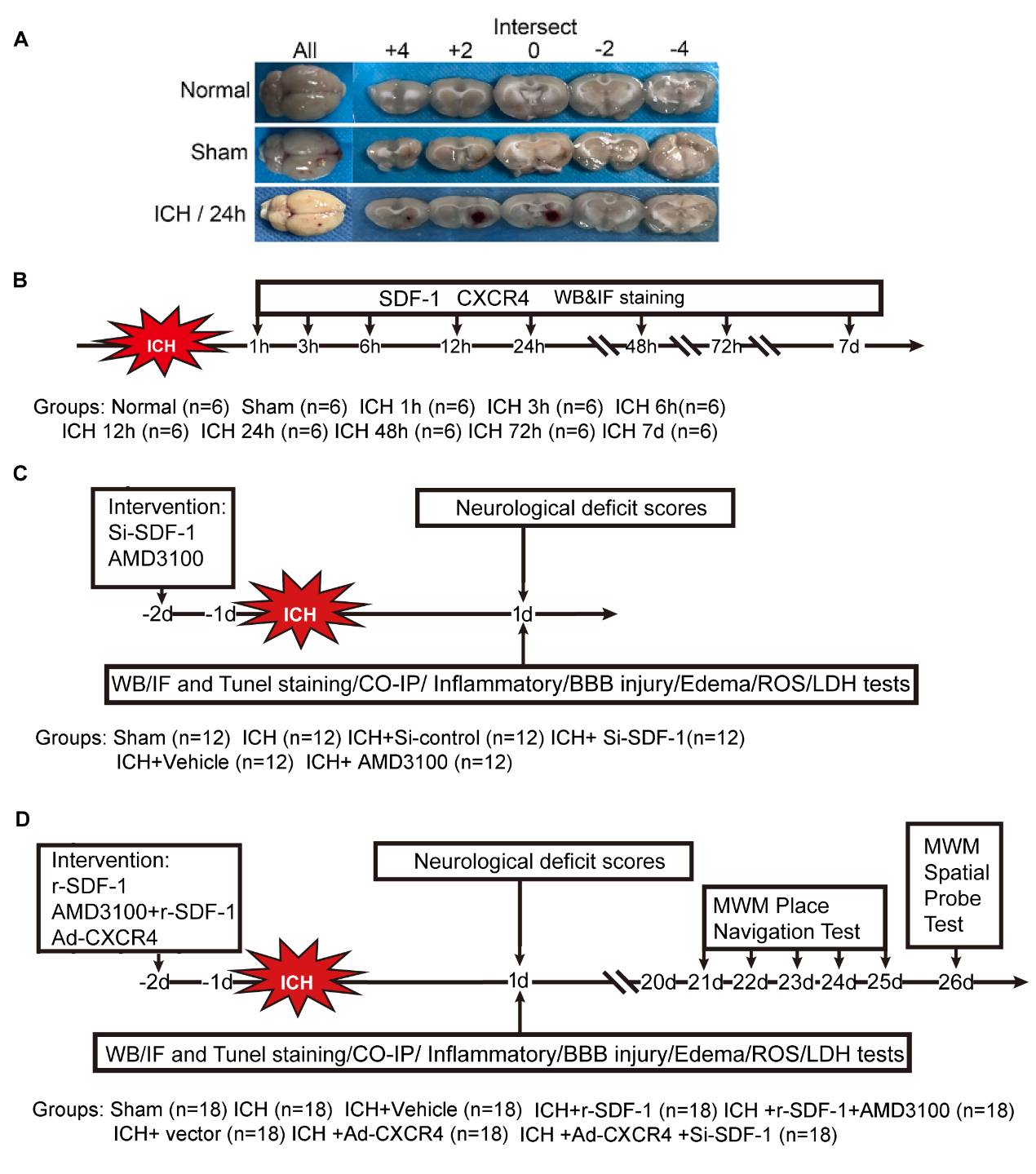


Figure S1. Intracerebral hemorrhage (ICH) model and experimental design. (A) The general morphology of ICH model specimens from the different experimental groups. (B) Time course of protein levels of SDF-1 and CXCR4 after ICH. (C) Knockdown of SDF-1 on ICH brain injury and its possible role. (D) The role of Overexpression of SDF-1 and CXCR4 in brain injury after ICH.


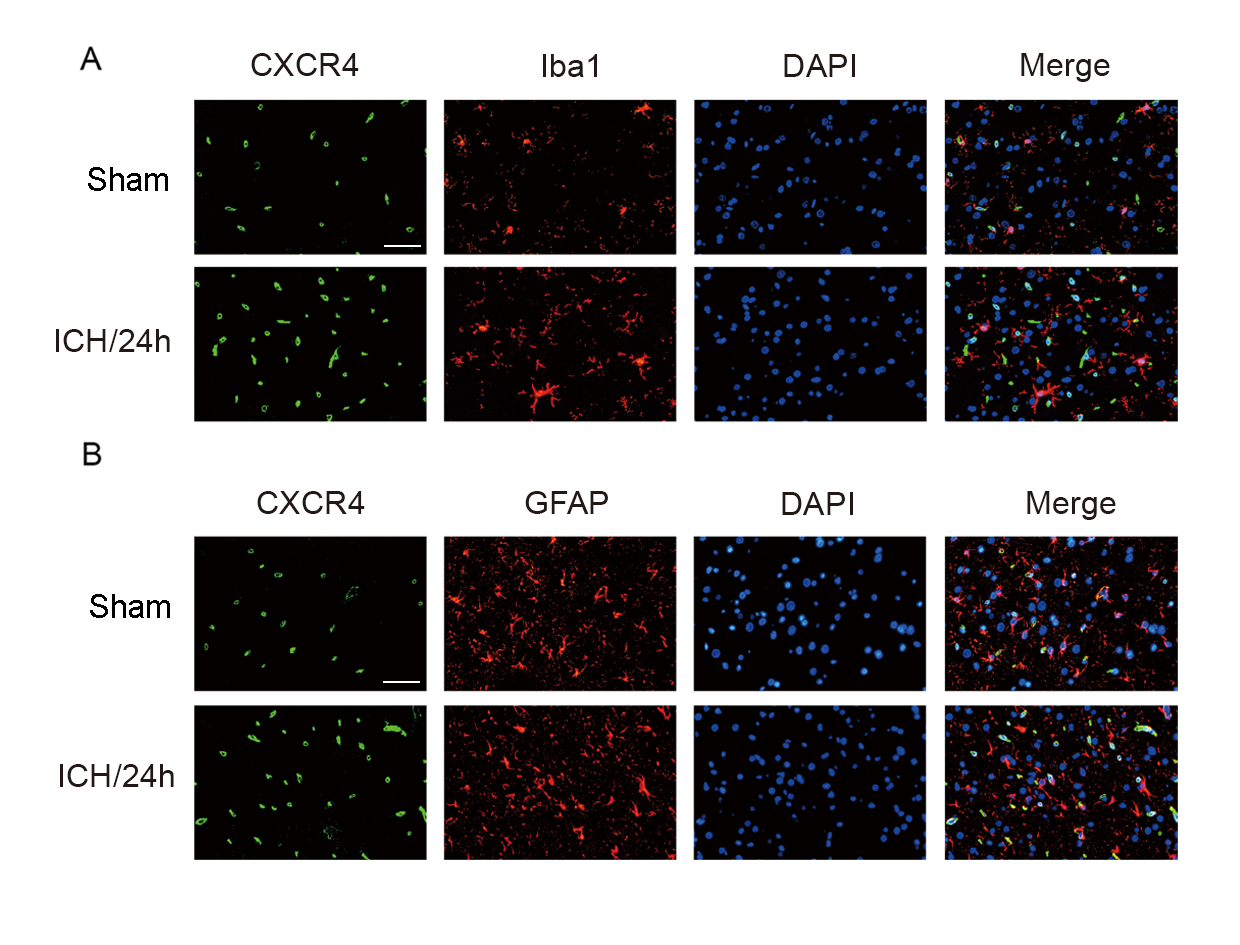


Figure S2. (A) The immunofluorescence results showed that CXCR4 (green), microglia marker (Iba1, red), and nuclei were fluorescently labeled with DAPI (blue) in brain tissue of rats in Sham group and ICH group. (B) The immunofluorescence results showed that CXCR4 (green), astrocyte marker (GFAP, red), and nuclei were fluorescently labeled with DAPI (blue) in brain tissue of rats in Sham group and ICH group. Scale bar=50 μm.


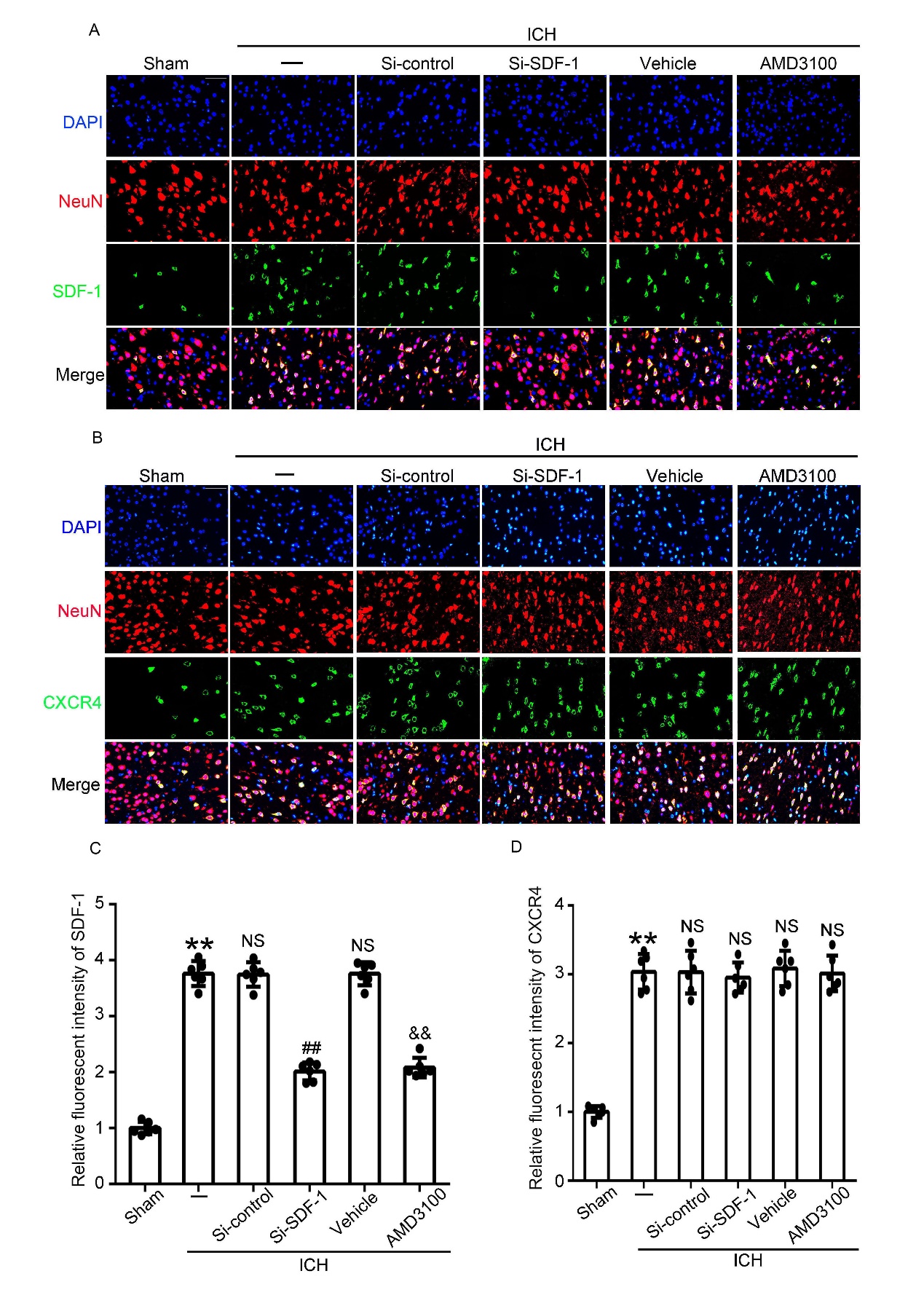


Figure S3. Immunofluorescence staining under conditions to change SDF-1 (A, C) and CXCR4 (B, D) expression after ICH. Green was the SDF-1/CXCR4 and red was NeuN, and Blue was DAPI. The brain regions used for slides were from the basal ganglia region of the right hemisphere of rats. Data are shown as the mean±SEM; **P < 0.01 vs. Sham; ##P < 0.05 vs. ICH+Si-control; &&P < 0.01 vs. ICH + Vehicle. Scale bar=50μm.


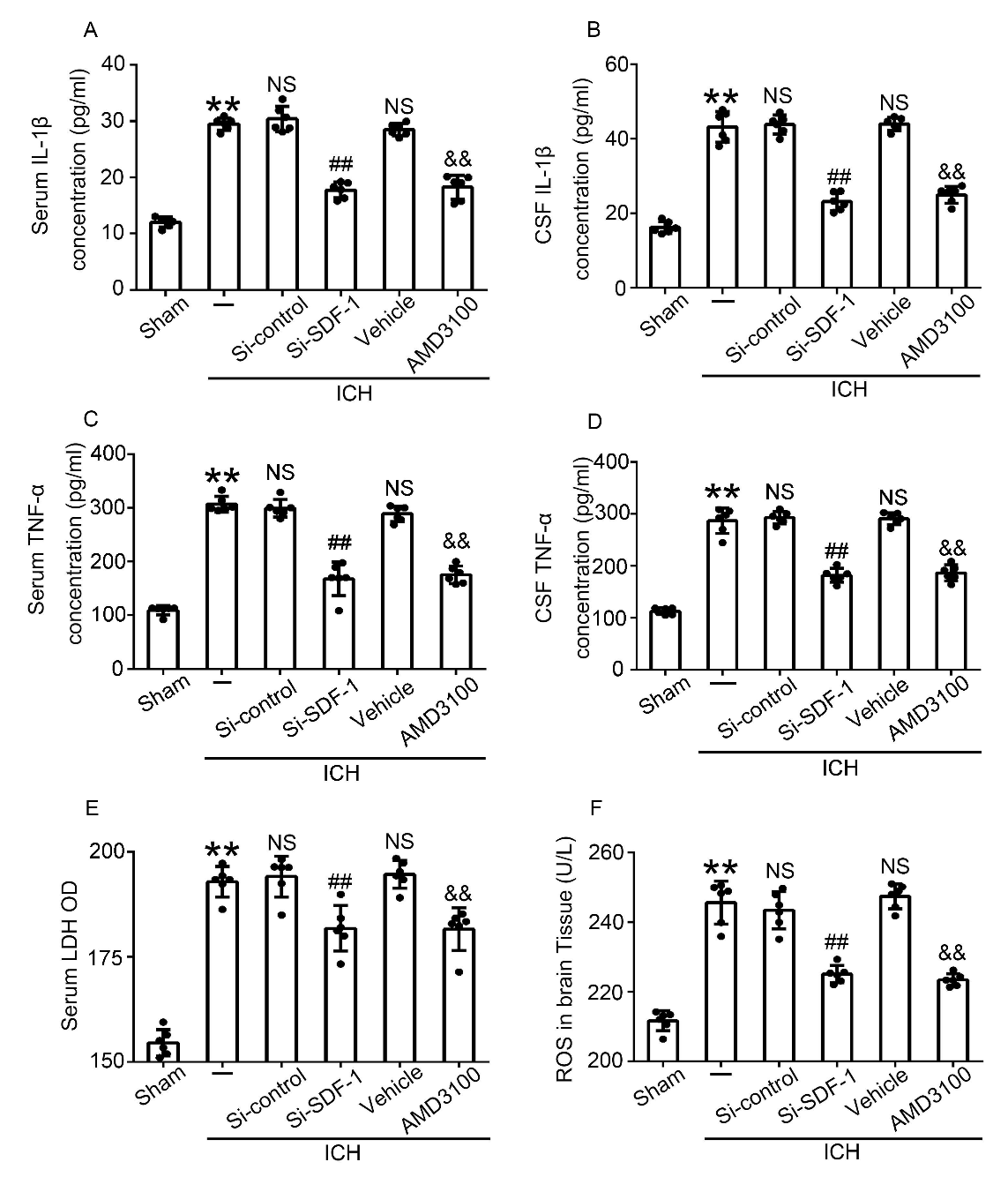


Figure S4. Analysis of CSF LDH levels and levels of inflammatory factors in serum and CSF of rats in various treatments groups (A-E). ROS levels were also measured in brain tissue of rats in various treatments groups (F). Data are shown as the mean±SEM; **P < 0.01 vs. Sham; ##P < 0.01 vs. ICH+ Si-control; &&P < 0.01 vs. ICH+ Vehicle.


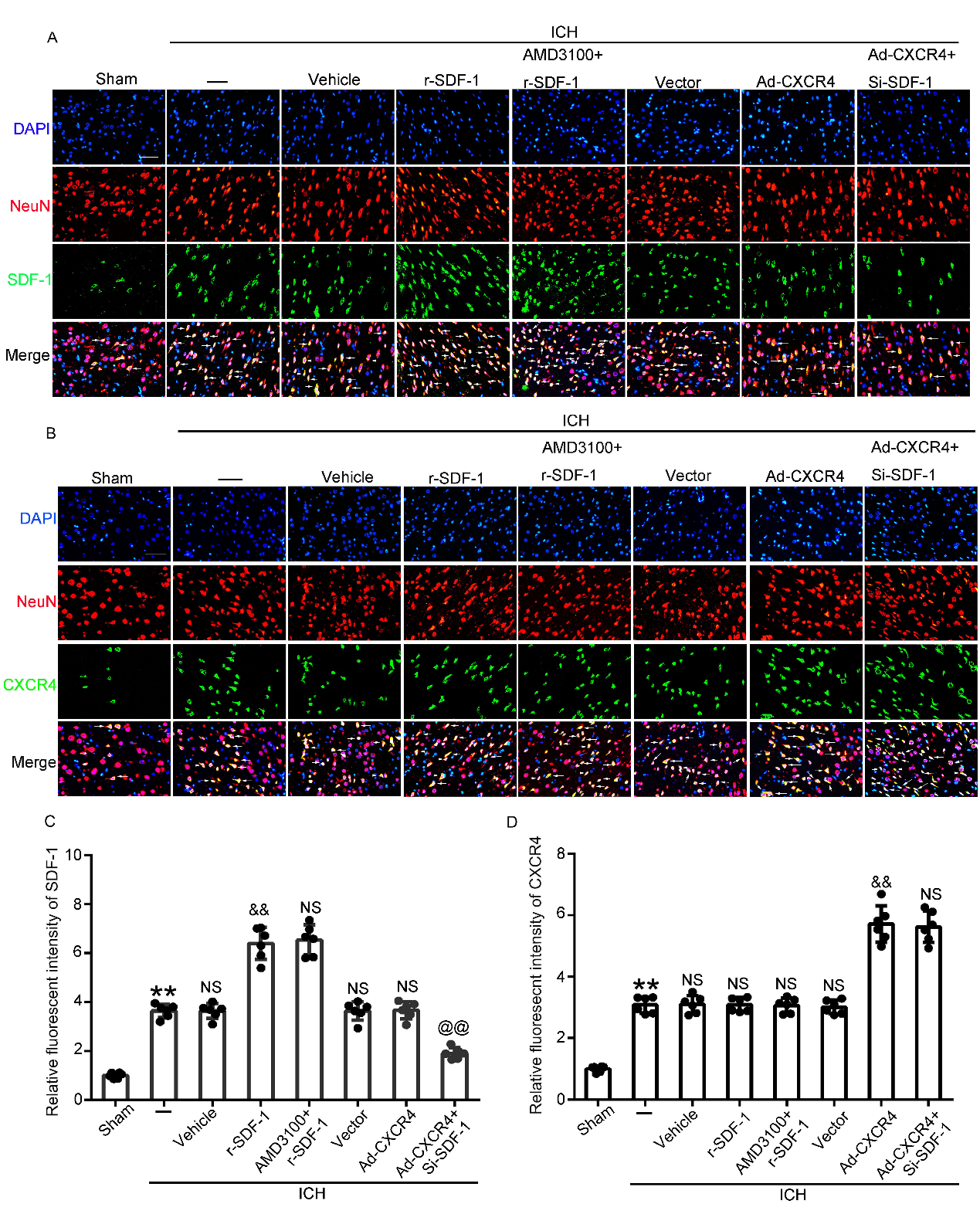


Figure S5. Immunofluorescence staining under conditions to increase SDF-1 (A, C) and CXCR4 (B, D) expression after ICH. Green was the SDF-1/CXCR4 and red was NeuN, and Blue was DAPI. The brain regions used for slides were from the basal ganglia region of the right hemisphere of rats. Data are shown as the mean±SEM; **P < 0.01 vs. Sham; &&P < 0.01 vs. ICH+Vehicle (in C); &&P < 0.01 vs. ICH+Vector (in D); @@P < 0.01 vs. ICH+Ad-CXCR4. Scale bar=50μm.


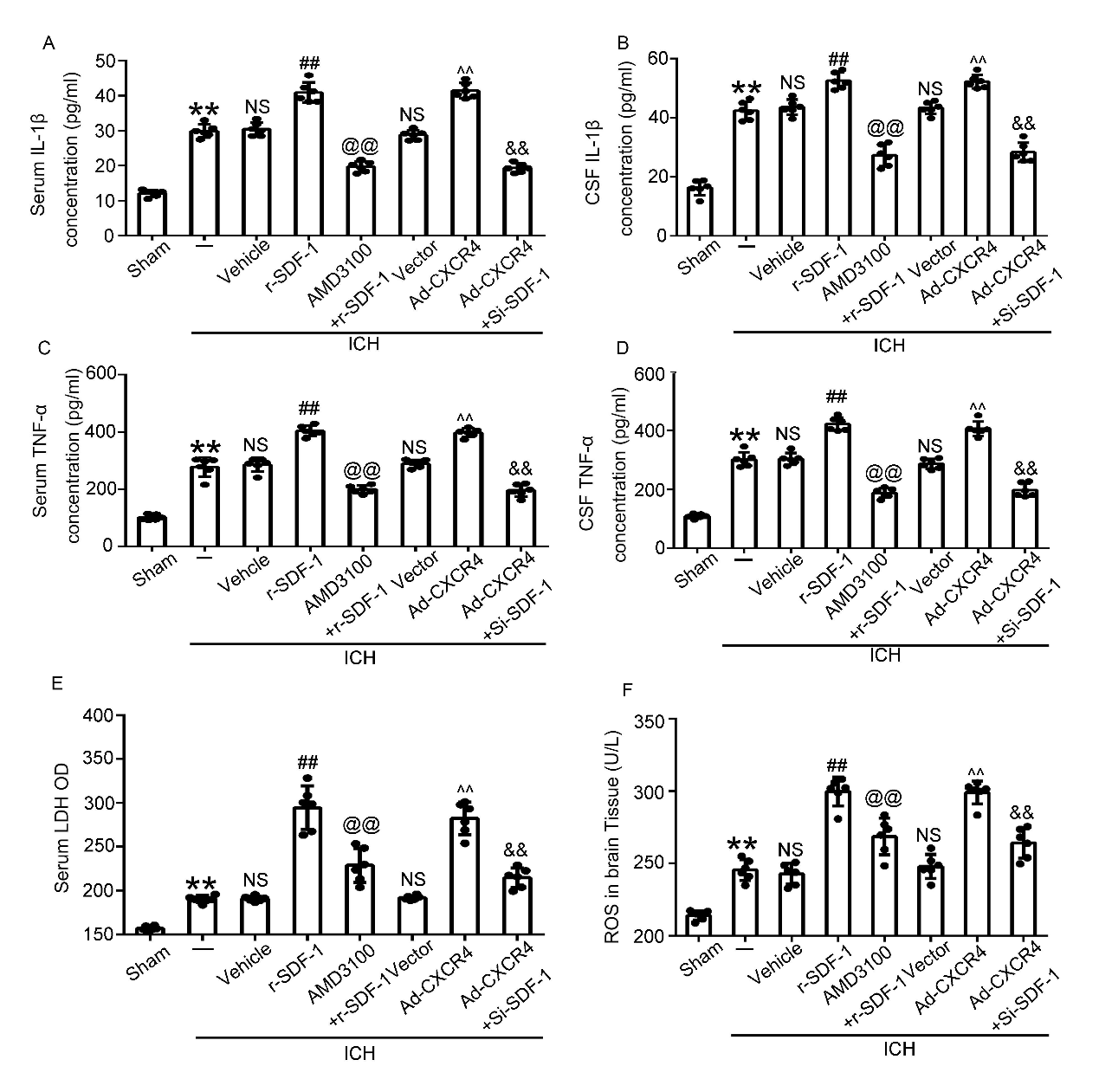


Figure S6. Analysis of CSF LDH levels and levels of inflammatory factors in serum and CSF of rats in various treatments groups involving overexpression of SDF-1 and/or CXCR4 (A-E). ROS levels were also measured in brain tissue of rats in each group (F). Data are shown as the mean±SEM; **P < 0.01 vs. Sham; ##P < 0.01 vs. ICH+Vehicle; @@P < 0.01 vs. ICH+r-SDF-1; ^^P < 0.01 vs. ICH+Vector; &&P < 0.01 vs. ICH+Ad-CXCR4.
