# Supplementary material for: Stromal cell‐derived factor‐1 downregulation contributes to neuroprotection mediated by CXC chemokine receptor 4 interactions after intracerebral hemorrhage in rats
Source: CNS Neurosci Ther. 2023 Aug 24;30(2):e14400. doi: 10.1111/cns.14400 (PMC10848108; doi:10.1111/cns.14400)

Full unedited gel/blot for Figure 1

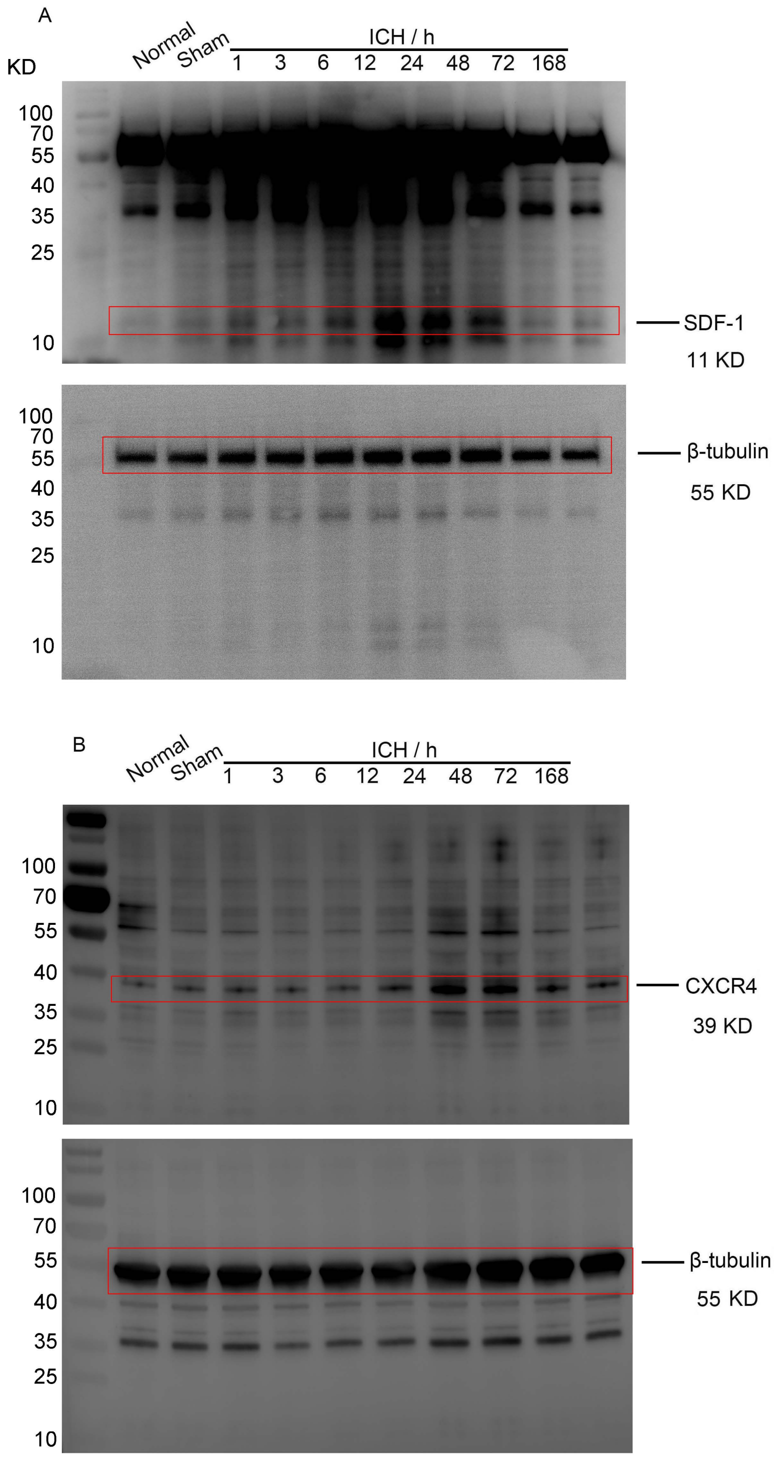

Full unedited gel/blot for Figure 2

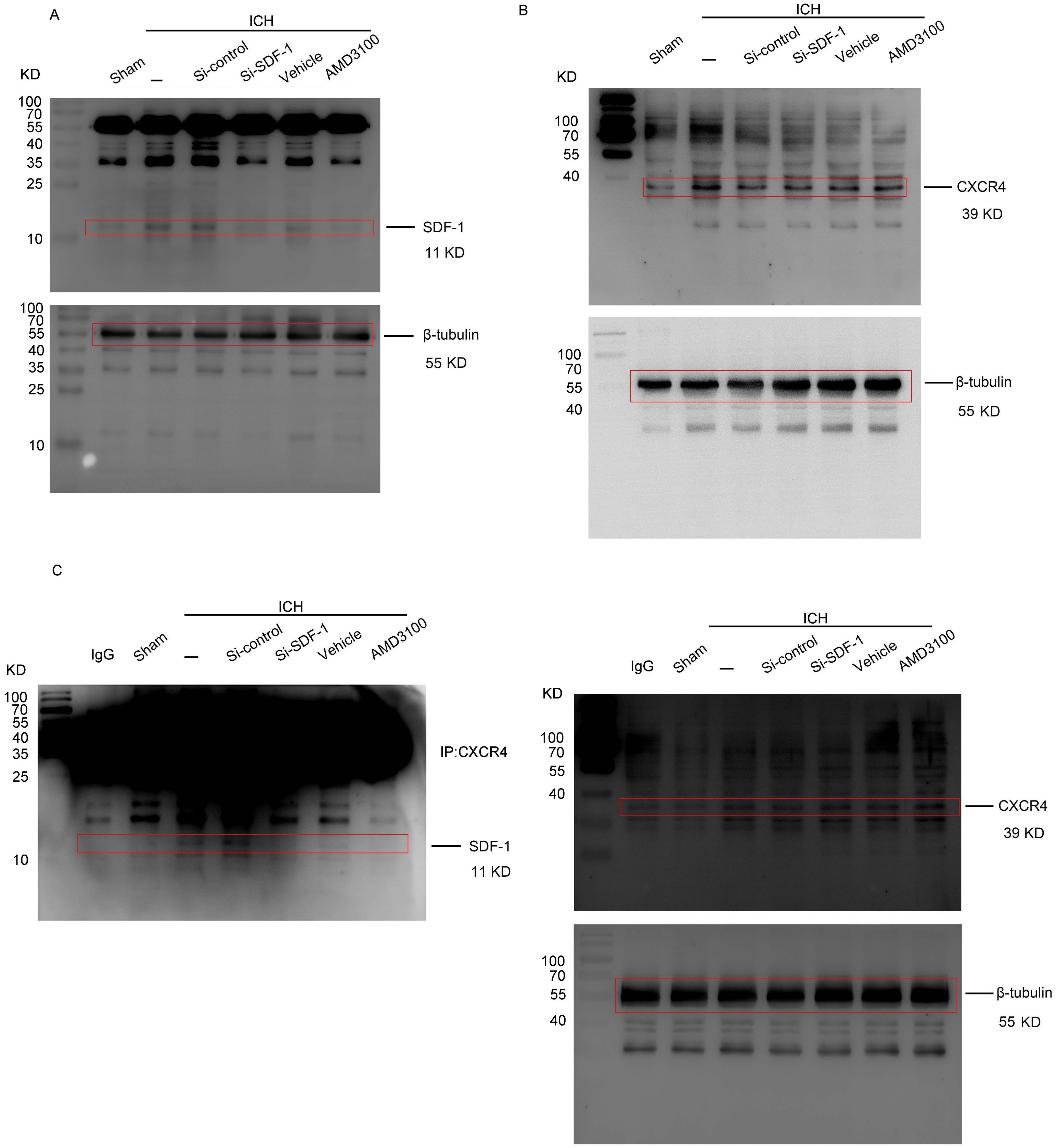

Full unedited gel/blot for Figure 3

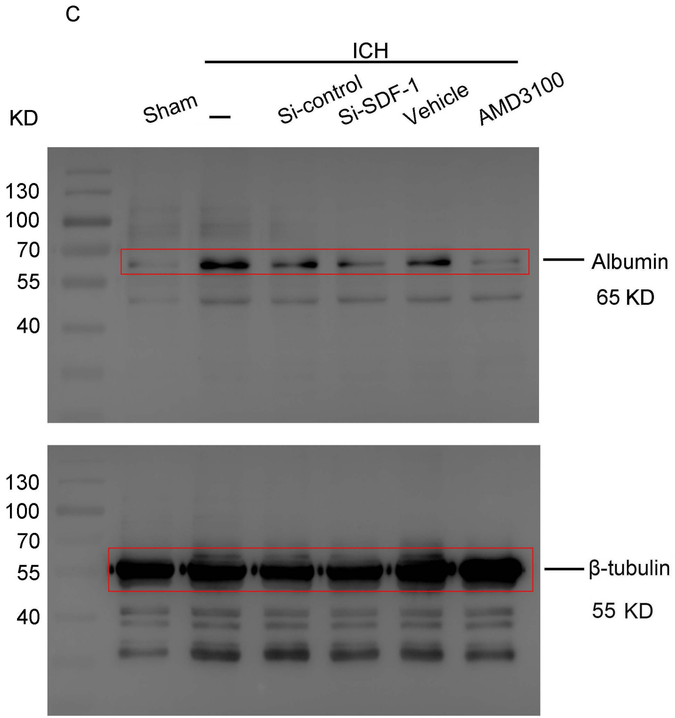

Full unedited gel/blot for Figure 4

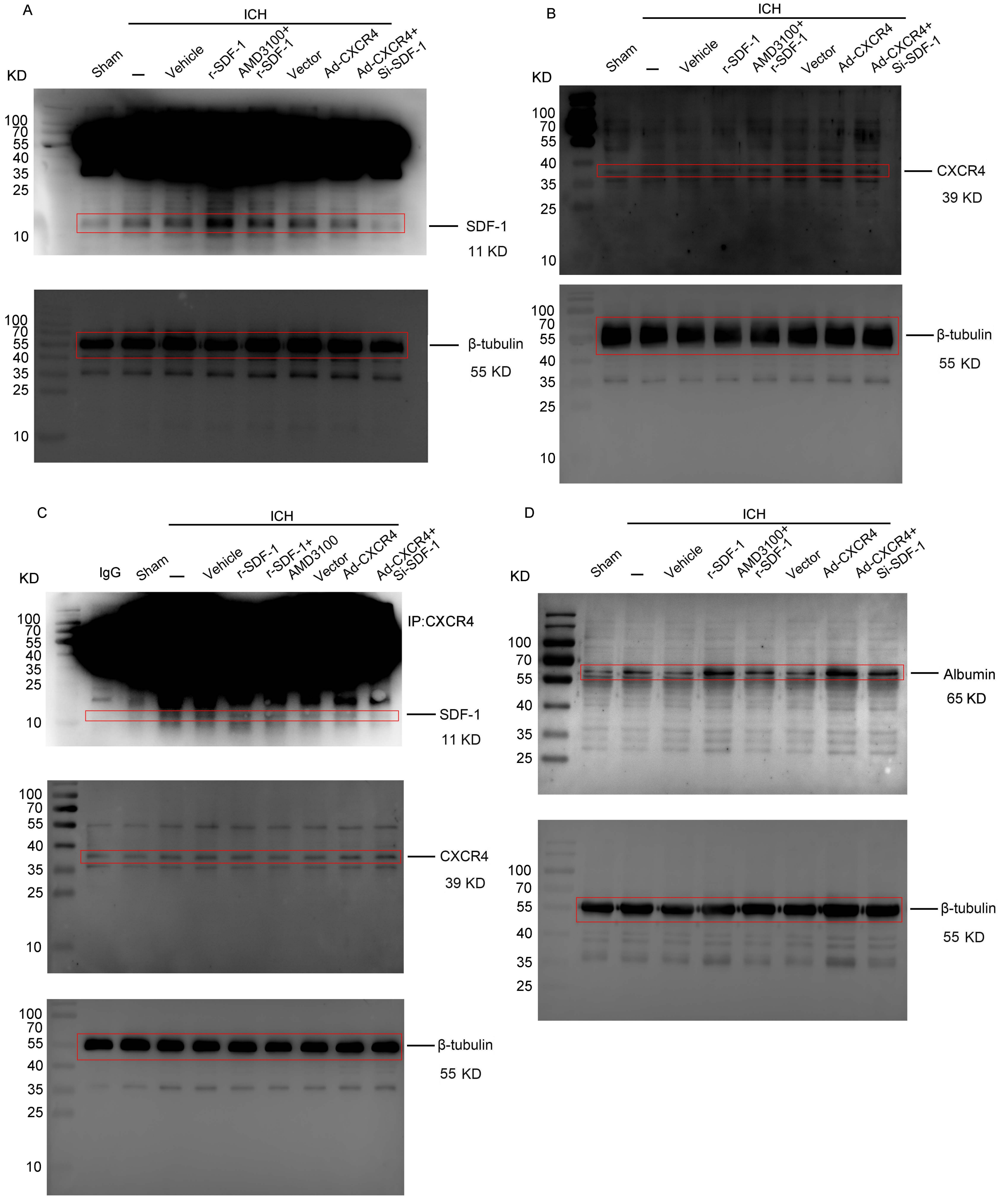

Supplement: Supplementary file 1 — Appendix S1. [file CNS-30-e14400-s001.zip › cns14400-sup-0002-supinfo.pdf]
