# Supplementary material for: Stromal cell‐derived factor‐1 downregulation contributes to neuroprotection mediated by CXC chemokine receptor 4 interactions after intracerebral hemorrhage in rats
Source: CNS Neurosci Ther. 2023 Aug 24;30(2):e14400. doi: 10.1111/cns.14400 (PMC10848108; doi:10.1111/cns.14400)
Supplement: Supplementary file 2 — Table S1. [file CNS-30-e14400-s002.docx]

**Table S1: Detailed statistical table**

| Fig. | Description | Test used | Statistical item | Stat-value |
| --- | --- | --- | --- | --- |
| 1A | Relative protein level of SDF1 | Unpaired t test | Sham vs. Normal | Difference between means ± SEM：0.04689 ± 0.05262；95% CI：-0.07035 to 0.1641；P=0.3938 |
|  |  |  | Sham vs. ICH 6h | Difference between means ± SEM：0.3731 ± 0.09196；95% CI：0.1682 to 0.5780；P=0.0023 |
|  |  |  | Sham vs. ICH 12h | Difference between means ± SEM：1.895 ± 0.07244；95% CI：1.734 to 2.057；P<0.0001 |
|  |  |  | Sham vs. ICH 24h | Difference between means ± SEM：2.060 ± 0.07156  ；95% CI：1.900 to 2.219；P<0.0001 |
|  |  |  | Sham vs. ICH 48h | Difference between means ± SEM：0.7888 ± 0.1143；95% CI：0.5342 to 1.043；P<0.0001 |
| 1B | Relative protein level of CXCR4 | Unpaired t test | Sham vs. Normal | Difference between means ± SEM：0.04806 ± 0.08311；95% CI：-0.1371 to 0.2332；P=0.5759 |
|  |  |  | Sham vs. ICH 24h | Difference between means ± SEM：1.587 ± 0.08956  ；95% CI：1.387 to 1.786；P<0.0001 |
|  |  |  | Sham vs. ICH 48h | Difference between means ± SEM：1.557 ± 0.1142；95% CI：1.303 to 1.811；P<0.0001 |
|  |  |  | Sham vs. ICH 72h | Difference between means ± SEM：0.5338 ± 0.1353；95% CI：0.2324 to 0.8352；P =0.0027 |
| 1C | Relative fluorescent intensity of SDF-1 | Unpaired t test | Sham vs. Normal | Difference between means ± SEM：0.01547 ± 0.06727；95% CI：-0.1344 to 0.1654；P=0.8228 |
|  |  |  | Sham vs. ICH 12h | Difference between means ± SEM：2.953 ± 0.06655；95% CI：2.805 to 3.101；P<0.0001 |
|  |  |  | Sham vs. ICH 24h | Difference between means ± SEM：2.914 ± 0.05468；95% CI：2.792 to 3.036；P<0.0001 |
| 1D | Relative fluorescent intensity of CXCR4 | Unpaired t test | Sham vs. Normal | Difference between means ± SEM：-0.009000 ± 0.06800；95% CI：-0.1605 to 0.1425；P=0.8973 |
|  |  |  | Sham vs. ICH 24h | Difference between means ± SEM：1.749 ± 0.1218；95% CI：1.477 to 2.020；P<0.0001 |
|  |  |  | Sham vs. ICH 48h | Difference between means ± SEM：1.651 ± 0.09498；95% CI：1.439 to 1.863；P<0.0001 |

| Fig. | Description | Test used | Statistical item | Stat-value |
| --- | --- | --- | --- | --- |
| 2A | Relative protein level of SDF1 | Unpaired t test | Sham vs. ICH | Difference between means ± SEM：3.533 ± 0.05803；95% CI：3.399 to 3.667；P<0.0001 |
|  |  |  | ICH vs. Si-control | Difference between means ± SEM：0.02482 ± 0.1017；95% CI：-0.2098 to 0.2594；P=0.8134 |
|  |  |  | Si-control vs. Si-SDF-1 | Difference between means ± SEM：-1.630 ± 0.1414；95% CI：-1.956 to -1.304；P<0.0001 |
|  |  |  | ICH vs. Vehicle | Difference between means ± SEM：-0.1437 ± 0.1119；95% CI：-0.4018 to 0.1144；P=0.2351 |
|  |  |  | Vehicle vs. AMD3100 | Difference between means ± SEM：-1.876 ± 0.2033  ；95% CI：-2.345 to -1.407；P<0.0001 |
| 2B | Relative protein level of CXCR4 | Unpaired t test | Sham vs. ICH | Difference between means ± SEM：1.820 ± 0.02976；95% CI：1.753 to 1.886；P<0.0001 |
|  |  |  | ICH vs. Si-control | Difference between means ± SEM：0.04432 ± 0.03258；95% CI：-0.02826 to 0.1169；P=0.2035 |
|  |  |  | Si-control vs. Si-SDF-1 | Difference between means ± SEM：-0.04289 ± 0.03214；95% CI：-0.1145 to 0.02872；P=0.2116 |
|  |  |  | ICH vs. Vehicle | Difference between means ± SEM：-0.005844 ± 0.03765；95% CI：-0.08973 to 0.07804；P=0.8797 |
|  |  |  | Vehicle vs. AMD3100 | Difference between means ± SEM：-0.001988 ± 0.04127；95% CI：-0.09393 to 0.08996；P=0.9625 |
| 2D | Relative density CXCR4/β-tubulin | Unpaired t test | Sham vs. ICH | Difference between means ± SEM：1.616 ± 0.09885；95% CI：1.396 to 1.837；P<0.0001 |
|  |  |  | ICH vs. Si-control | Difference between means ± SEM：-0.1819 ± 0.1291；95% CI：-0.4695 to 0.1056；P=0.1890 |
|  |  |  | Si-control vs. Si-SDF-1 | Difference between means ± SEM：0.3123 ± 0.1695；95% CI：-0.06532 to 0.6900；P=0.0952 |
|  |  |  | ICH vs. Vehicle | Difference between means ± SEM：0.2483 ± 0.1829  ；95% CI：-0.1593 to 0.6558；P=0.2045 |
|  |  |  | Vehicle vs. AMD3100 | Difference between means ± SEM：-0.2647 ± 0.1839  ；95% CI：-0.6744 to 0.1451；P=0.1807 |
| 2E | Relative density SDF-1/CXCR4 | Unpaired t test | Sham vs. ICH | Difference between means ± SEM：2.609 ± 0.1955；95% CI：2.174 to 3.045；P<0.0001 |
|  |  |  | ICH vs. Si-control | Difference between means ± SEM：0.1535 ± 0.2752；95% CI：-0.4596 to 0.7665；P=0.5893 |
|  |  |  | Si-control vs. Si-SDF-1 | Difference between means ± SEM：-1.856 ± 0.2427  ；95% CI：-2.397 to -1.315；P<0.0001 |
|  |  |  | ICH vs. Vehicle | Difference between means ± SEM：-0.2028 ± 0.2473；95% CI：-0.7538 to 0.3482；P=0.4313 |
|  |  |  | Vehicle vs. AMD3100 | Difference between means ± SEM：-1.666 ± 0.1792；95% CI：-2.066 to -1.267；P<0.0001 |
| 2F | Neuroscore | Unpaired t test | Sham vs. ICH | Difference between means ± SEM：-8.000 ± 0.5676；95% CI：-9.265 to -6.735；P<0.0001 |
|  |  |  | ICH vs. Si-control | Difference between means ± SEM：-0.5000 ± 0.5821；95% CI：-1.797 to 0.7971；P=0.4105 |
|  |  |  | Si-control vs. Si-SDF-1 | Difference between means ± SEM：4.167 ± 0.6872；95% CI：2.636 to 5.698；P=0.0001 |
|  |  |  | ICH vs. Vehicle | Difference between means ± SEM：-0.5000 ± 0.5821；95% CI：-1.797 to 0.7971；P=0.4105 |
|  |  |  | Vehicle vs. AMD3100 | Difference between means ± SEM：3.667 ± 0.8300；95% CI：1.817 to 5.516；P=0.0013 |
| 3B | Precentage of TUNEL-positive cell | Unpaired t test | Sham vs. ICH | Difference between means ± SEM：16.00 ± 0.6992；95% CI：14.44 to 17.56；P<0.0001 |
|  |  |  | ICH vs. Si-control | Difference between means ± SEM：0.000 ± 1.011；95% CI：-2.253 to 2.253；P>0.9999 |
|  |  |  | Si-control vs. Si-SDF-1 | Difference between means ± SEM：-8.833 ± 0.8724；95% CI：-10.78 to -6.889；P<0.0001 |
|  |  |  | ICH vs. Vehicle | Difference between means ± SEM：-0.1667 ± 1.035；95% CI：-2.474 to 2.141；P=0.8753 |
|  |  |  | Vehicle vs. AMD3100 | Difference between means ± SEM：-9.000 ± 0.9944；95% CI：-11.22 to -6.784；P<0.0001 |
| 3D | Relative protein levels of Albumin | Unpaired t test | Sham vs. ICH | Difference between means ± SEM：16.16 ± 0.8612；95% CI：14.25 to 18.08；P<0.0001 |
|  |  |  | ICH vs. Si-control | Difference between means ± SEM：1.179 ± 1.161；95% CI：-1.406 to 3.765；P=0.3334 |
|  |  |  | Si-control vs. Si-SDF-1 | Difference between means ± SEM：-11.44 ± 0.8153；95% CI：-13.26 to -9.624；P<0.0001 |
|  |  |  | ICH vs. Vehicle | Difference between means ± SEM：-0.5826 ± 1.087；95% CI：-3.004 to 1.839；P=0.6036 |
|  |  |  | Vehicle vs. AMD3100 | Difference between means ± SEM：-10.96 ± 1.048；95% CI：-13.30 to -8.626；P<0.0001 |
| 3E | Brain water content | Two-way ANOVA | Sham vs. ICH | Difference between means ± SEM：  -0.008344 ± 0.0006327；95% CI：-0.01017 to -0.006517；P<0.0001 |
|  |  |  | ICH vs. Si-control | Difference between means ± SEM：0.0008063± 0.0006327；95% CI：-0.001020 to 0.002633；P=0.7985 |
|  |  |  | Si-control vs. Si-SDF-1 | Difference between means ± SEM：0.003677± 0.0006327；95% CI：0.001850 to 0.005503  ；P<0.0001 |
|  |  |  | ICH vs. Vehicle | Difference between means ± SEM：-5.600e-006± 0.0006327；95% CI：-0.001832 to 0.001821  ；P>0.9999 |
|  |  |  | Vehicle vs. AMD3100 | Difference between means ± SEM：0.004871± 0.0006327；95% CI：0.003045 to 0.006698；P<0.0001 |
| S3C | Relative fluorescent intensity of SDF-1 | Unpaired t test | Sham vs. ICH | Difference between means ± SEM：2.762 ± 0.1015；95% CI：2.536 to 2.988；P<0.0001 |
|  |  |  | ICH vs. Si-control | Difference between means ± SEM：-0.01669 ± 0.1273；95% CI：-0.3004 to 0.2670；P=0.8983 |
|  |  |  | Si-control vs. Si-SDF-1 | Difference between means ± SEM：-1.733 ± 0.1109；95% CI：-1.980 to -1.486；P<0.0001 |
|  |  |  | ICH vs. Vehicle | Difference between means ± SEM：-0.003416 ± 0.1237；95% CI：-0.2791 to 0.2722；P=0.9785 |
|  |  |  | Vehicle vs. AMD3100 | Difference between means ± SEM：-1.677 ± 0.1102；95% CI：-1.923 to -1.432；P<0.0001 |
| S3D | Relative fluoresent intensitv of CXCR4 | Unpaired t test | Sham vs. ICH | Difference between means ± SEM：2.036 ± 0.1115；95% CI：1.788 to 2.285；P<0.0001 |
|  |  |  | ICH vs. Si-control | Difference between means ± SEM：-0.005346 ± 0.1648；95% CI：-0.3725 to 0.3618；P=0.9748 |
|  |  |  | Si-control vs. Si-SDF-1 | Difference between means ± SEM：-0.07828 ± 0.1545；95% CI：-0.4225 to 0.2659；P=0.6234 |
|  |  |  | ICH vs. Vehicle | Difference between means ± SEM：0.04925 ± 0.1487；95% CI：-0.2820 to 0.3805；P=0.7473 |
|  |  |  | Vehicle vs. AMD3100 | Difference between means ± SEM：-0.07296 ± 0.1486；95% CI：-0.4041 to 0.2582；P=0.6341 |
| S4A | Serum IL-1β concentration | Unpaired t test | Sham vs. ICH | Difference between means ± SEM：17.47 ± 0.5776；95% CI：16.18 to 18.75；P<0.0001 |
|  |  |  | ICH vs. Si-control | Difference between means ± SEM：0.8747 ± 1.009；95% CI：-1.374 to 3.124；P=0.4064 |
|  |  |  | Si-control vs. Si-SDF-1 | Difference between means ± SEM：-12.63 ± 1.071；95% CI：-15.02 to -10.25；P<0.0001 |
|  |  |  | ICH vs. Vehicle | Difference between means ± SEM：-0.9972 ± 0.6245；95% CI：-2.389 to 0.3943；P=0.1414 |
|  |  |  | Vehicle vs. AMD3100 | Difference between means ± SEM：-10.26 ± 0.9758；95% CI：-12.43 to -8.086；P<0.0001 |
| S4B | CSF IL-1β concentration | Unpaired t test | Sham vs. ICH | Difference between means ± SEM：26.95 ± 1.804；95% CI：22.93 to 30.97；P<0.0001 |
|  |  |  | ICH vs. Si-control | Difference between means ± SEM：0.6525 ± 1.969；95% CI：-3.736 to 5.041；P=0.7472 |
|  |  |  | Si-control vs. Si-SDF-1 | Difference between means ± SEM：-20.73 ± 1.420；95% CI：-23.89 to -17.56；P<0.0001 |
|  |  |  | ICH vs. Vehicle | Difference between means ± SEM：0.7722 ± 1.824；95% CI：-3.291 to 4.835；P=0.6809 |
|  |  |  | Vehicle vs. AMD3100 | Difference between means ± SEM：-19.02 ± 1.163；95% CI：-21.61 to -16.42；P<0.0001 |
| S4C | Serum TNF-α concentration | Unpaired t test | Sham vs. ICH | Difference between means ± SEM：197.5 ± 6.845；95% CI：182.2 to 212.7；P<0.0001 |
|  |  |  | ICH vs. Si-control | Difference between means ± SEM：-7.472 ± 8.924；95% CI：-27.36 to 12.41；P=0.4220 |
|  |  |  | Si-control vs. Si-SDF-1 | Difference between means ± SEM：-131.7 ± 14.38；95% CI：-163.7 to -99.66；P<0.0001 |
|  |  |  | ICH vs. Vehicle | Difference between means ± SEM：-18.03 ± 8.350；95% CI：-36.64 to 0.5696；P=0.0561 |
|  |  |  | Vehicle vs. AMD3100 | Difference between means ± SEM：-113.3 ± 8.888；95% CI：-133.1 to -93.52；P<0.0001 |
| S4D | CSF TNF-α concentration | Unpaired t test | Sham vs. ICH | Difference between means ± SEM：174.1 ± 10.37；95% CI：151.0 to 197.2；P<0.0001 |
|  |  |  | ICH vs. Si-control | Difference between means ± SEM：6.150 ± 11.15；95% CI：-18.69 to 30.99；P=0.5933 |
|  |  |  | Si-control vs. Si-SDF-1 | Difference between means ± SEM：-111.2 ± 7.348；95% CI：-127.6 to -94.83；P<0.0001 |
|  |  |  | ICH vs. Vehicle | Difference between means ± SEM：4.108 ± 11.08；95% CI：-20.57 to 28.79；P=0.7185 |
|  |  |  | Vehicle vs. AMD3100 | Difference between means ± SEM：-104.5 ± 7.895；95% CI：-122.1 to -86.95；P<0.0001 |
| S4E | Serum LDH OD | Unpaired t test | Sham vs. ICH | Difference between means ± SEM：38.28 ± 1.954；95% CI：33.93 to 42.64；P<0.0001 |
|  |  |  | ICH vs. Si-control | Difference between means ± SEM：1.272 ± 2.473；95% CI：-4.239 to 6.783；P=0.6182 |
|  |  |  | Si-control vs. Si-SDF-1 | Difference between means ± SEM：-12.34 ± 2.972；95% CI：-18.97 to -5.721；P=0.0020 |
|  |  |  | ICH vs. Vehicle | Difference between means ± SEM：1.803 ± 2.004；95% CI：-2.662 to 6.268；P=0.3894 |
|  |  |  | Vehicle vs. AMD3100 | Difference between means ± SEM：-13.05 ± 2.475；95% CI：-18.57 to -7.535；P=0.0004 |
| S4F | ROS in brain tissue | Unpaired t test | Sham vs. ICH | Difference between means ± SEM：33.96 ± 2.768；95% CI：27.80 to 40.13；P<0.0001 |
|  |  |  | ICH vs. Si-control | Difference between means ± SEM：-2.186 ± 3.327；95% CI：-9.600 to 5.228；P=0.5261 |
|  |  |  | Si-control vs. Si-SDF-1 | Difference between means ± SEM：-18.36 ± 2.412；95% CI：-23.74 to -12.98；P<0.0001 |
|  |  |  | ICH vs. Vehicle | Difference between means ± SEM：1.788 ± 2.903；95% CI：-4.679 to 8.256；P=0.5516 |
|  |  |  | Vehicle vs. AMD3100 | Difference between means ± SEM：-24.00 ± 1.625；95% CI：-27.62 to -20.37；P<0.0001 |

| Fig. | Description | Test used | Statistical item | Stat-value |
| --- | --- | --- | --- | --- |
| 4A | Relative protein levels of SDF1 | Unpaired t test | Sham vs. ICH | Difference between means ± SEM：2.691 ± 0.1647；95% CI：2.324 to 3.058；P<0.0001 |
|  |  |  | ICH vs. Vehicle | Difference between means ± SEM：0.007902 ± 0.1575；95% CI：-0.3430 to 0.3588；P=0.9610 |
|  |  |  | Vehicle vs. r-SDF-1 | Difference between means ± SEM：2.615 ± 0.2387；95% CI：2.083 to 3.147；P<0.0001 |
|  |  |  | r-SDF-1 vs. AMD3100+ r-SDF-1 | Difference between means ± SEM：0.3585 ± 0.3398；95% CI：-0.3987 to 1.116；P=0.3163 |
|  |  |  | ICH vs. Vector | Difference between means ± SEM：-0.1737 ± 0.1933；95% CI：-0.6043 to 0.2569；P=0.3898 |
|  |  |  | Vector vs. Ad-CXCR4 | Difference between means ± SEM：0.08864 ± 0.1781；95% CI：-0.3083 to 0.4855；P=0.6295 |
|  |  |  | Ad-CXCR4 vs. Ad-CXCR4+Si-SDF-1 | Difference between means ± SEM：-1.632 ± 0.1956；95% CI：-2.068 to -1.196；P<0.0001 |
| 4B | Relative protein levels of CXCR4 | Unpaired t test | Sham vs. ICH | Difference between means ± SEM：1.845 ± 0.07915；95% CI：1.669 to 2.021；P<0.0001 |
|  |  |  | ICH vs. Vehicle | Difference between means ± SEM：0.06121 ± 0.08144；95% CI：-0.1202 to 0.2427；P=0.4696 |
|  |  |  | Vehicle vs. r-SDF-1 | Difference between means ± SEM：0.1040 ± 0.09275；95% CI：-0.1026 to 0.3107；P=0.2882 |
|  |  |  | r-SDF-1 vs. AMD3100+ r-SDF-1 | Difference between means ± SEM：-0.1807 ± 0.1310；95% CI：-0.4725 to 0.1111；P=0.1976 |
|  |  |  | ICH vs. Vector | Difference between means ± SEM：-0.05444 ± 0.1974；95% CI：-0.4943 to 0.3855；P=0.7884 |
|  |  |  | Vector vs. Ad-CXCR4 | Difference between means ± SEM：3.928 ± 0.2625；95% CI：3.343 to 4.513；P<0.0001 |
|  |  |  | Ad-CXCR4 vs. Ad-CXCR4+Si-SDF-1 | Difference between means ± SEM：0.1372 ± 0.2566；95% CI：-0.4345 to 0.7090；P=0.6045 |
| 4E | Relative protein levels of SDF-1/CXCR4 | Unpaired t test | Sham vs. ICH | Difference between means ± SEM：2.778 ± 0.2683；95% CI：2.181 to 3.376；P<0.0001 |
|  |  |  | ICH vs. Vehicle | Difference between means ± SEM：0.0001745 ± 0.2937；95% CI：-0.6543 to 0.6546；P=0.9995 |
|  |  |  | Vehicle vs. r-SDF-1 | Difference between means ± SEM：1.946 ± 0.3530；95% CI：1.159 to 2.732；P=0.0003 |
|  |  |  | r-SDF-1 vs. AMD3100+ r-SDF-1 | Difference between means ± SEM：-3.431 ± 0.3711；95% CI：-4.258 to -2.604；P<0.0001 |
|  |  |  | ICH vs. Vector | Difference between means ± SEM：-0.5800 ± 0.2920；95% CI：-1.231 to 0.07062；P=0.0751 |
|  |  |  | Vector vs. Ad-CXCR4 | Difference between means ± SEM：2.227 ± 0.2884；95% CI：1.584 to 2.869；P<0.0001 |
|  |  |  | Ad-CXCR4 vs. Ad-CXCR4+Si-SDF-1 | Difference between means ± SEM：-3.197 ± 0.2563；95% CI：-3.768 to -2.626；P<0.0001 |
| 4F | Relative protein levels of CXCR4/β-tubulin | Unpaired t test | Sham vs. ICH | Difference between means ± SEM：1.100 ± 0.1551；95% CI：0.7548 to 1.446；P<0.0001 |
|  |  |  | ICH vs. Vehicle | Difference between means ± SEM：0.06952 ± 0.1687；95% CI：-0.3064 to 0.4455；P=0.6890 |
|  |  |  | Vehicle vs. r-SDF-1 | Difference between means ± SEM：0.1626 ± 0.1587；95% CI：-0.1910 to 0.5163；P=0.3297 |
|  |  |  | r-SDF-1 vs. AMD3100+ r-SDF-1 | Difference between means ± SEM：-0.3910 ± 0.1891；95% CI：-0.8123 to 0.03026；P=0.0655 |
|  |  |  | ICH vs. Vector | Difference between means ± SEM：0.04542 ± 0.2157；95% CI：-0.4352 to 0.5261；P=0.8375 |
|  |  |  | Vector vs. Ad-CXCR4 | Difference between means ± SEM：2.312 ± 0.3278；95% CI：1.581 to 3.042；P<0.0001 |
|  |  |  | Ad-CXCR4 vs. Ad-CXCR4+Si-SDF-1 | Difference between means ± SEM：0.4319 ± 0.3757；95% CI：-0.4051 to 1.269；P=0.2770 |
| 4G | Relative protein levels of Albumin | Unpaired t test | Sham vs. ICH | Difference between means ± SEM：7.829 ± 0.2019；95% CI：7.379 to 8.279；P<0.0001 |
|  |  |  | ICH vs. Vehicle | Difference between means ± SEM：0.01813 ± 0.5111；95% CI：-1.121 to 1.157；P=0.9742 |
|  |  |  | Vehicle vs. r-SDF-1 | Difference between means ± SEM：7.161 ± 0.7207；95% CI：5.555 to 8.767；P<0.0001 |
|  |  |  | r-SDF-1 vs. AMD3100+ r-SDF-1 | Difference between means ± SEM：-8.903 ± 0.6796；95% CI：-10.42 to -7.389；P<0.0001 |
|  |  |  | ICH vs. Vector | Difference between means ± SEM：0.6165 ± 0.4831；95% CI：-0.4598 to 1.693；P=0.2307 |
|  |  |  | Vector vs. Ad-CXCR4 | Difference between means ± SEM：6.338 ± 0.6861；95% CI：4.809 to 7.867；P<0.0001 |
|  |  |  | Ad-CXCR4 vs. Ad-CXCR4+Si-SDF-1 | Difference between means ± SEM：-8.431 ± 0.5964；95% CI：-9.759 to -7.102；P<0.0001 |
| 5B | Precentage of TUNEL-positive cell | Unpaired t test | Sham vs. ICH | Difference between means ± SEM：14.50 ± 0.6368；95% CI：13.08 to 15.92；P<0.0001 |
|  |  |  | ICH vs. Vehicle | Difference between means ± SEM：0.8333 ± 0.9690；95% CI：-1.326 to 2.992；P=0.4099 |
|  |  |  | Vehicle vs. r-SDF-1 | Difference between means ± SEM：3.833 ± 1.249；95% CI：1.049 to 6.617；P=0.0119 |
|  |  |  | r-SDF-1 vs. AMD3100+ r-SDF-1 | Difference between means ± SEM：-11.33 ± 1.160；95% CI：-13.92 to -8.750；P<0.0001 |
|  |  |  | ICH vs. Vector | Difference between means ± SEM：0.5000 ± 0.7782  ；95% CI：-1.234 to 2.234；P=0.5350 |
|  |  |  | Vector vs. Ad-CXCR4 | Difference between means ± SEM：4.833 ± 0.7782；95% CI：3.099 to 6.567；P<0.0001 |
|  |  |  | Ad-CXCR4 vs. Ad-CXCR4+Si-SDF-1 | Difference between means ± SEM：-12.33 ± 0.8498；95% CI：-14.23 to -10.44；P<0.0001 |
| 5C | Neuroscore | Unpaired t test | Sham vs. ICH | Difference between means ± SEM：-7.883 ± 0.5426；95% CI：-9.042 to -6.624；P<0.0001 |
|  |  |  | ICH vs. Vehicle | Difference between means ± SEM：0.0 ± 0.6055；95% CI：-1.349 to 1.349；P>0.9999 |
|  |  |  | Vehicle vs. r-SDF-1 | Difference between means ± SEM：-4.667 ± 0.5270；95% CI：-5.841 to -3.492；P<0.0001 |
|  |  |  | r-SDF-1 vs. AMD3100+ r-SDF-1 | Difference between means ± SEM：2.167 ± 0.4773；95% CI：1.103 to 3.230；P=0.0011 |
|  |  |  | ICH vs. Vector | Difference between means ± SEM：-0.1667 ± 0.6009；95% CI：-1.506 to 1.172；P=0.7872 |
|  |  |  | Vector vs. Ad-CXCR4 | Difference between means ± SEM：-4.500 ± 0.5217；95% CI：-5.663 to -3.337；P<0.0001 |
|  |  |  | Ad-CXCR4 vs. Ad-CXCR4+Si-SDF-1 | Difference between means ± SEM：2.333 ± 0.4346；95% CI：1.365 to 3.302；P=0.0003 |
| 5D | Brain water content | Two-way ANOVA | Sham vs. ICH | Difference between means ± SEM：-0.007362± 0.0005820；95% CI：-0.009145 to -0.005579；P<0.0001 |
|  |  |  | ICH vs. Vehicle | Difference between means ± SEM：0.0003800± 0.0005820；95% CI：-0.001403 to 0.002163；P=0.9980 |
|  |  |  | Vehicle vs. r-SDF-1 | Difference between means ± SEM：-0.003901± 0.0005820；95% CI：-0.005684 to -0.002118；P<0.0001 |
|  |  |  | r-SDF-1 vs. AMD3100+ r-SDF-1 | Difference between means ± SEM：0.007575± 0.0005820；95% CI：0.005793 to 0.009358；P<0.0001 |
|  |  |  | ICH vs. Vector | Difference between means ± SEM：-0.0003357± 0.0005820；95% CI：-0.002119 to 0.001447；P=  0.9991 |
|  |  |  | Vector vs. Ad-CXCR4 | Difference between means ± SEM：-0.003729± 0.0005820；95% CI：-0.005512 to -0.001947；P<0.0001 |
|  |  |  | Ad-CXCR4 vs. Ad-CXCR4+Si-SDF-1 | Difference between means ± SEM：0.007922± 0.0005820；95% CI：0.006139 to 0.009705；P<0.0001 |
| 6A | Swimming Distance | Two-way ANOVA | Sham vs. ICH | Difference between means ± SEM：-330.8± 31.94；95% CI：-428.7 to -233.0；P<0.0001 |
|  |  |  | ICH vs. Vehicle | Difference between means ± SEM：80.10± 31.94；95% CI：-17.76 to 178.0；P=0.1983 |
|  |  |  | Vehicle vs. r-SDF-1 | Difference between means ± SEM：-263.7± 31.94；95% CI：-361.6 to -165.9；P<0.0001 |
|  |  |  | r-SDF-1 vs. AMD3100+ r-SDF-1 | Difference between means ± SEM：168.5± 31.94；95% CI：70.60 to 266.3；P<0.0001 |
|  |  |  | ICH vs. Vector | Difference between means ± SEM：-34.76± 31.94；95% CI：-132.6 to 63.09；P=0.9587 |
|  |  |  | Vector vs. Ad-CXCR4 | Difference between means ± SEM：-125.0± 31.94；95% CI：-222.9 to -27.17；P=0.0031 |
|  |  |  | Ad-CXCR4 vs. Ad-CXCR4+Si-SDF-1 | Difference between means ± SEM：201.7± 31.94；95% CI：103.8 to 299.5；P<0.0001 |
| 6B | Water Mase Latency | Two-way ANOVA | Sham vs. ICH | Difference between means ± SEM：-10.10± 0.8382；95% CI：-12.66 to -7.528；P<0.0001 |
|  |  |  | ICH vs. Vehicle | Difference between means ± SEM：1.065± 0.8382；95% CI：-1.503 to 3.633；P=0.9086 |
|  |  |  | Vehicle vs. r-SDF-1 | Difference between means ± SEM：-8.313± 0.8382；95% CI：-10.88 to -5.745；P<0.0001 |
|  |  |  | r-SDF-1 vs. AMD3100+ r-SDF-1 | Difference between means ± SEM：5.792± 0.8382；95% CI：3.224 to 8.360；P<0.0001 |
|  |  |  | ICH vs. Vector | Difference between means ± SEM：-1.945± 0.8382；95% CI：-4.513 to 0.6225；P=0.2876 |
|  |  |  | Vector vs. Ad-CXCR4 | Difference between means ± SEM：-4.915± 0.8382；95% CI：-7.483 to -2.348；P<0.0001 |
|  |  |  | Ad-CXCR4 vs. Ad-CXCR4+Si-SDF-1 | Difference between means ± SEM：7.420± 0.8382；95% CI：4.852 to 9.988；P<0.0001 |
| 6C | Average Speed | Unpaired t test | Sham vs. ICH | Difference between means ± SEM：0.6890 ± 0.6642；95% CI：-0.7909 to 2.169；P=0.3240 |
|  |  |  | ICH vs. Vehicle | Difference between means ± SEM：-1.254 ± 0.7308；95% CI：-2.882 to 0.3746；P=0.1170 |
|  |  |  | Vehicle vs. r-SDF-1 | Difference between means ± SEM：0.6583 ± 0.8391；95% CI：-1.211 to 2.528；P=0.4509 |
|  |  |  | r-SDF-1 vs. AMD3100+ r-SDF-1 | Difference between means ± SEM：0.03900 ± 1.001；95% CI：-2.192 to 2.270；P=0.9697 |
|  |  |  | ICH vs. Vector | Difference between means ± SEM：-0.5207 ± 0.8406；95% CI：-2.394 to 1.352；P=0.5495 |
|  |  |  | Vector vs. Ad-CXCR4 | Difference between means ± SEM：-0.5960 ± 1.342；95% CI：-3.586 to 2.394；P=0.6664 |
|  |  |  | Ad-CXCR4 vs. Ad-CXCR4+Si-SDF-1 | Difference between means ± SEM：0.8943 ± 1.490；95% CI：-2.426 to 4.215；P=0.5617 |
| 6D | Time in target quadrant | Unpaired t test | Sham vs. ICH | Difference between means ± SEM：-0.1387 ± 0.01866；95% CI：-0.1803 to -0.09712；P<0.0001 |
|  |  |  | ICH vs. Vehicle | Difference between means ± SEM：0.001392 ± 0.01579；95% CI：-0.03379 to 0.03658；P=0.9315 |
|  |  |  | Vehicle vs. r-SDF-1 | Difference between means ± SEM：0.06845 ± 0.02054；95% CI：0.02268 to 0.1142；P=0.0076 |
|  |  |  | r-SDF-1 vs. AMD3100+ r-SDF-1 | Difference between means ± SEM：-0.1221 ± 0.02020；95% CI：-0.1671 to -0.07707；P=0.0001 |
|  |  |  | ICH vs. Vector | Difference between means ± SEM：0.01690 ± 0.01284；95% CI：-0.01170 to 0.04550；P=0.2174 |
|  |  |  | Vector vs. Ad-CXCR4 | Difference between means ± SEM：0.06916 ± 0.01459；95% CI：0.03664 to 0.1017；P=0.0008 |
|  |  |  | Ad-CXCR4 vs. Ad-CXCR4+Si-SDF-1 | Difference between means ± SEM：-0.1464 ± 0.01529；95% CI：-0.1805 to -0.1123；P<0.0001 |
| S5C | Relative fluorescent intensity of SDF-1 | Unpaired t test | Sham vs. ICH | Difference between means ± SEM：2.639 ± 0.1213；95% CI：2.368 to 2.909；P<0.0001 |
|  |  |  | ICH vs. Vehicle | Difference between means ± SEM：-0.001969 ± 0.1688；95% CI：-0.3781 to 0.3742；P=0.9909 |
|  |  |  | Vehicle vs. r-SDF-1 | Difference between means ± SEM：2.762 ± 0.2950；95% CI：2.104 to 3.419；P<0.0001 |
|  |  |  | r-SDF-1 vs. AMD3100+ r-SDF-1 | Difference between means ± SEM：0.1371 ± 0.3660；95% CI：-0.6785 to 0.9526；P=0.7159 |
|  |  |  | ICH vs. Vector | Difference between means ± SEM：-0.0004980 ± 0.1915；95% CI：-0.4272 to 0.4262；P=0.9980 |
|  |  |  | Vector vs. Ad-CXCR4 | Difference between means ± SEM：0.03303 ± 0.2118；95% CI：-0.4388 to 0.5049；P=0.8792 |
|  |  |  | Ad-CXCR4 vs. Ad-CXCR4+Si-SDF-1 | Difference between means ± SEM：-1.767 ± 0.1716；95% CI：-2.150 to -1.385；P<0.0001 |
| S5D | Relative fluoresent intensity of CXCR4 | Unpaired t test | Sham vs. ICH | Difference between means ± SEM：2.081 ± 0.1149；95% CI：1.825 to 2.337；P<0.0001 |
|  |  |  | ICH vs. Vehicle | Difference between means ± SEM：0.02068 ± 0.1581；95% CI：-0.3315 to 0.3729；P=0.8985 |
|  |  |  | Vehicle vs. r-SDF-1 | Difference between means ± SEM：-0.01383 ± 0.1485；95% CI：-0.3448 to 0.3171；P=0.9277 |
|  |  |  | r-SDF-1 vs. AMD3100+ r-SDF-1 | Difference between means ± SEM：-0.02202 ± 0.1349；95% CI：-0.3225 to 0.2785；P=0.8736 |
|  |  |  | ICH vs. Vector | Difference between means ± SEM：-0.06750 ± 0.1407；95% CI：-0.3809 to 0.2459；P=0.6416 |
|  |  |  | Vector vs. Ad-CXCR4 | Difference between means ± SEM：2.706 ± 0.2584；95% CI：2.130 to 3.282；P<0.0001 |
|  |  |  | Ad-CXCR4 vs. Ad-CXCR4+Si-SDF-1 | Difference between means ± SEM：-0.09381 ± 0.3189；95% CI：-0.8043 to 0.6166；P=0.7746 |
| S6A | Serum IL-1β concentration | Unpaired t test | Sham vs. ICH | Difference between means ± SEM：17.87 ± 0.8634；95% CI：15.94 to 19.79；P<0.0001 |
|  |  |  | ICH vs. Vehicle | Difference between means ± SEM：0.5371 ± 1.098；95% CI：-1.909 to 2.983；P=0.6352 |
|  |  |  | Vehicle vs. r-SDF-1 | Difference between means ± SEM：10.46 ± 1.424；95% CI：7.288 to 13.63；P<0.0001 |
|  |  |  | r-SDF-1 vs. AMD3100+ r-SDF-1 | Difference between means ± SEM：-21.18 ± 1.341；95% CI：-24.17 to -18.19；P<0.0001 |
|  |  |  | ICH vs. Vector | Difference between means ± SEM：-1.145 ± 0.9504；95% CI：-3.263 to 0.9727；P=0.2561 |
|  |  |  | Vector vs. Ad-CXCR4 | Difference between means ± SEM：12.68 ± 1.056；95% CI：10.33 to 15.04；P<0.0001 |
|  |  |  | Ad-CXCR4 vs. Ad-CXCR4+Si-SDF-1 | Difference between means ± SEM：-22.08 ± 1.040；95% CI：-24.40 to -19.76；P<0.0001 |
| S6B | CSF IL-1β concentration | Unpaired t test | Sham vs. ICH | Difference between means ± SEM：26.05 ± 1.634；95% CI：22.41 to 29.69；P<0.0001 |
|  |  |  | ICH vs. Vehicle | Difference between means ± SEM：1.156 ± 1.644；95% CI：-2.506 to 4.818；P=0.4979 |
|  |  |  | Vehicle vs. r-SDF-1 | Difference between means ± SEM：9.007 ± 1.545；95% CI：5.565 to 12.45；P=0.0002 |
|  |  |  | r-SDF-1 vs. AMD3100+ r-SDF-1 | Difference between means ± SEM：-25.24 ± 1.871；95% CI：-29.41 to -21.07；P<0.0001 |
|  |  |  | ICH vs. Vector | Difference between means ± SEM：0.9626 ± 1.518；95% CI：-2.420 to 4.345；P=0.5403 |
|  |  |  | Vector vs. Ad-CXCR4 | Difference between means ± SEM：8.889 ± 1.282；95% CI：6.033 to 11.75；P<0.0001 |
|  |  |  | Ad-CXCR4 vs. Ad-CXCR4+Si-SDF-1 | Difference between means ± SEM：-23.96 ± 1.630；95% CI：-27.59 to -20.32；P<0.0001 |
| S6C | Serum TNF-α concentration | Unpaired t test | Sham vs. ICH | Difference between means ± SEM：176.2 ± 14.20；95% CI：144.5 to 207.8；P<0.0001 |
|  |  |  | ICH vs. Vehicle | Difference between means ± SEM：8.054 ± 16.61；95% CI：-28.96 to 45.07；P=0.6382 |
|  |  |  | Vehicle vs. r-SDF-1 | Difference between means ± SEM：117.9 ± 12.12；95% CI：90.89 to 144.9；P<0.0001 |
|  |  |  | r-SDF-1 vs. AMD3100+ r-SDF-1 | Difference between means ± SEM：-204.6 ± 9.273；95% CI：-225.3 to -183.9；P<0.0001 |
|  |  |  | ICH vs. Vector | Difference between means ± SEM：11.29 ± 14.73；95% CI：-21.52 to 44.11；P=0.4609 |
|  |  |  | Vector vs. Ad-CXCR4 | Difference between means ± SEM：108.2 ± 8.258；95% CI：89.82 to 126.6；P<0.0001 |
|  |  |  | Ad-CXCR4 vs. Ad-CXCR4+Si-SDF-1 | Difference between means ± SEM：-201.2 ± 10.60；95% CI：-224.8 to -177.6；P<0.0001 |
| S6D | CSF TNF-α concentration | Unpaired t test | Sham vs. ICH | Difference between means ± SEM：192.5 ± 10.63；95% CI：168.8 to 216.2；P<0.0001 |
|  |  |  | ICH vs. Vehicle | Difference between means ± SEM：2.200 ± 12.99；95% CI：-26.75 to 31.15；P=0.8689 |
|  |  |  | Vehicle vs. r-SDF-1 | Difference between means ± SEM：118.2 ± 12.22；95% CI：90.93 to 145.4；P<0.0001 |
|  |  |  | r-SDF-1 vs. AMD3100+ r-SDF-1 | Difference between means ± SEM：-233.9 ± 11.33；95% CI：-259.2 to -208.7；P<0.0001 |
|  |  |  | ICH vs. Vector | Difference between means ± SEM：-14.53 ± 12.29；95% CI：-41.90 to 12.85；P=0.2644 |
|  |  |  | Vector vs. Ad-CXCR4 | Difference between means ± SEM：118.8 ± 12.28；95% CI：91.41 to 146.2；P<0.0001 |
|  |  |  | Ad-CXCR4 vs. Ad-CXCR4+Si-SDF-1 | Difference between means ± SEM：-207.9 ± 13.87；95% CI：-238.8 to -177.0；P<0.0001 |
| S6E | Serum LDH OD | Unpaired t test | Sham vs. ICH | Difference between means ± SEM：33.66 ± 1.951；95% CI：29.31 to 38.00；P<0.0001 |
|  |  |  | ICH vs. Vehicle | Difference between means ± SEM：0.06703 ± 2.028；95% CI：-4.451 to 4.585；P=0.9743 |
|  |  |  | Vehicle vs. r-SDF-1 | Difference between means ± SEM：103.8 ± 10.20；95% CI：81.07 to 126.5；P<0.0001 |
|  |  |  | r-SDF-1 vs. AMD3100+ r-SDF-1 | Difference between means ± SEM：-65.70 ± 12.85；95% CI：-94.34 to -37.06；P=0.0005 |
|  |  |  | ICH vs. Vector | Difference between means ± SEM：1.301 ± 1.902；95% CI：-2.938 to 5.540；P=0.5096 |
|  |  |  | Vector vs. Ad-CXCR4 | Difference between means ± SEM：90.42 ± 7.600  ；95% CI：73.48 to 107.4；P<0.0001 |
|  |  |  | Ad-CXCR4 vs. Ad-CXCR4+Si-SDF-1 | Difference between means ± SEM：-67.61 ± 8.805；95% CI：-87.23 to -48.00；P<0.0001 |
| S6F | ROS in brain tissue | Unpaired t test | Sham vs. ICH | Difference between means ± SEM：31.46 ± 3.272；95% CI：24.17 to 38.75；P<0.0001 |
|  |  |  | ICH vs. Vehicle | Difference between means ± SEM：-2.715 ± 4.195；95% CI：-12.06 to 6.633；P=0.5321 |
|  |  |  | Vehicle vs. r-SDF-1 | Difference between means ± SEM：56.87 ± 4.981；95% CI：45.77 to 67.97；P<0.0001 |
|  |  |  | r-SDF-1 vs. AMD3100+ r-SDF-1 | Difference between means ± SEM：-31.06 ± 6.546；95% CI：-45.64 to -16.47；P=0.0008 |
|  |  |  | ICH vs. Vector | Difference between means ± SEM：2.432 ± 4.535；95% CI：-7.672 to 12.54；P=0.6035 |
|  |  |  | Vector vs. Ad-CXCR4 | Difference between means ± SEM：51.03 ± 4.697；95% CI：40.56 to 61.49；P<0.0001 |
|  |  |  | Ad-CXCR4 vs. Ad-CXCR4+Si-SDF-1 | Difference between means ± SEM：-34.80 ± 5.354；95% CI：-46.73 to -22.87；P<0.0001 |
